# White matter microstructure and functional connectivity in the brains of infants with Turner syndrome

**DOI:** 10.1093/cercor/bhae351

**Published:** 2024-09-10

**Authors:** Reid Blanchett, Haitao Chen, Roza M Vlasova, Emil Cornea, Maria Maza, Marsha Davenport, Debra Reinhartsen, Margaret DeRamus, Rebecca Edmondson Pretzel, John H Gilmore, Stephen R Hooper, Martin A Styner, Wei Gao, Rebecca C Knickmeyer

**Affiliations:** Genetics and Genome Sciences, Michigan State University, Biomedical & Physical Sciences, Room 2165, East Lansing, MI 48824, United States; Department of Epigenetics, Van Andel Research Institute, 33 Bostwick Ave NE, Grand Rapids, MI 49503, United States; Biomedical Imaging Research Institute, Department of Biomedical Sciences and Imaging, 8700 Beverly Blvd, Cedars-Sinai Medical Center, Los Angeles, CA 90048, United States; Department of Psychiatry, 333 S. Columbia Street, Suite 304 MacNider Hall, University of North Carolina at Chapel Hill, Chapel Hill, NC 27514, United States; Department of Psychiatry, 333 S. Columbia Street, Suite 304 MacNider Hall, University of North Carolina at Chapel Hill, Chapel Hill, NC 27514, United States; Department of Psychology and Neuroscience, Campus Box #3270, 235 E. Cameron Avenue, University of North Carolina at Chapel Hill, Chapel Hill, NC 27599, United States; Department of Pediatrics, 333 South Columbia Street, Suite 260 MacNider Hall, University of North Carolina at Chapel Hill, Chapel Hill, NC 27599, United States; Carolina Institute for Developmental Disabilities, University of North Carolina at Chapel Hill, 101 Renee Lynn Ct, Carrboro, NC 27510, United States; Carolina Institute for Developmental Disabilities, University of North Carolina at Chapel Hill, 101 Renee Lynn Ct, Carrboro, NC 27510, United States; Carolina Institute for Developmental Disabilities, University of North Carolina at Chapel Hill, 101 Renee Lynn Ct, Carrboro, NC 27510, United States; Department of Psychiatry, 333 S. Columbia Street, Suite 304 MacNider Hall, University of North Carolina at Chapel Hill, Chapel Hill, NC 27514, United States; Department of Psychiatry, 333 S. Columbia Street, Suite 304 MacNider Hall, University of North Carolina at Chapel Hill, Chapel Hill, NC 27514, United States; Department of Health Sciences, Bondurant Hall, University of North Carolina at Chapel Hill, Chapel Hill, NC 27599, United States; Department of Psychiatry, 333 S. Columbia Street, Suite 304 MacNider Hall, University of North Carolina at Chapel Hill, Chapel Hill, NC 27514, United States; Department of Computer Science, Campus Box 3175, Brooks Computer Science Building, University of North Carolina at Chapel Hill, Chapel Hill, NC 27599, United States; Biomedical Imaging Research Institute, Department of Biomedical Sciences and Imaging, 8700 Beverly Blvd, Cedars-Sinai Medical Center, Los Angeles, CA 90048, United States; Department of Pediatrics and Human Development, Life Sciences Bldg. 1355 Bogue, #B240B, Michigan State University, East Lansing, MI 48824, United States; Institute for Quantitative Health Sciences and Engineering, Room 2114, 775 Woodlot Dr., East Lansing, MI 48824, United States

**Keywords:** diffusion tensor imaging, infancy, resting-state functional mri, Turner syndrome, X chromosome

## Abstract

Turner syndrome, caused by complete or partial loss of an X-chromosome, is often accompanied by specific cognitive challenges. Magnetic resonance imaging studies of adults and children with Turner syndrome suggest these deficits reflect differences in anatomical and functional connectivity. However, no imaging studies have explored connectivity in infants with Turner syndrome. Consequently, it is unclear when in development connectivity differences emerge. To address this gap, we compared functional connectivity and white matter microstructure of 1-year-old infants with Turner syndrome to typically developing 1-year-old boys and girls. We examined functional connectivity between the right precentral gyrus and five regions that show reduced volume in 1-year old infants with Turner syndrome compared to controls and found no differences. However, exploratory analyses suggested infants with Turner syndrome have altered connectivity between right supramarginal gyrus and left insula and right putamen. To assess anatomical connectivity, we examined diffusivity indices along the superior longitudinal fasciculus and found no differences. However, an exploratory analysis of 46 additional white matter tracts revealed significant group differences in nine tracts. Results suggest that the first year of life is a window in which interventions might prevent connectivity differences observed at later ages, and by extension, some of the cognitive challenges associated with Turner syndrome.

## Introduction

Described by Henry Turner in 1938 (TURNER 1938), Turner syndrome (TS) is caused by the partial or complete loss of an X-chromosome. The condition occurs in approximately 1 in 2,000 live female births (Nielsen and Wohlert 1991), making it one of the most common aneuploidies. TS represents a unique population for studying X chromosome effects on human development (Xie et al. 2017; Zhao and Gong 2017; Green et al. 2018), because females with TS are hemizygous for many genes in the pseudoautosomal regions (PARs) of the X chromosome, when compared to both XX females and XY males (Raznahan et al. 2018). For the 15% of genes outside the PAR that escape X-inactivation (Balaton et al. 2015), females with TS are expected to have reduced gene dosage compared to XX females, but similar dosage to XY males. The loss of the second sex chromosome produces multisystemic effects. TS is often accompanied by gonadal dysgenesis, congenital heart defects, renal abnormalities, and liver disorders (Gravholt et al. 2017), as well as a unique neurocognitive profile, particularly during the childhood and adolescent years. The latter profile has been described as comprising deficits in social cognition (SC) (Hong et al. 2009, 2011; Burnett et al. 2010), executive functioning (EF) (Kirk et al. 2005; Bray et al. 2011; Mauger et al. 2018), and visuospatial reasoning (VR) (Haberecht et al. 2001; Hart et al. 2006). Furthermore, individuals with TS appear to be at increased risk for male-biased neurodevelopmental disorders including autism spectrum disorders (ASDs) (Creswell and Skuse 1999; Avdic et al. 2021; Wolstencroft et al. 2022) and attention-deficit hyperactivity disorder (ADHD) (Green et al. 2018).

Structural magnetic resonance imaging (MRI) has been used to identify neuroanatomical features of TS that may explain the unique cognitive profile and increased risk for male-biased neurodevelopmental disorders. To date, most neuroimaging studies of TS have been performed on adults and adolescents who show structural changes consistent with the observed cognitive challenges: for example, decreased gray matter volume in right-sided parieto-occipital regions implicated in visuospatial reasoning and related nonverbal abilities (Brown et al. 2002; Molko et al. 2004; Cutter et al. 2006; Holzapfel et al. 2006; Marzelli et al. 2011). Consistently, white matter volume increases in TS have been observed in the temporal lobe, which is implicated in language and SC (Molko et al. 2004; Cutter et al. 2006; Holzapfel et al. 2006). The first quantitative neuroimaging study of infants with TS observed many structural features consistent with those present in adolescents and adults with TS, suggesting that many of the neuroanatomical phenotypes in TS are established early and persist into adulthood (Davenport et al. 2020).

High-level cognitive processes disrupted in TS, such as EF and SC, require orchestrated neurodevelopment and ultimately coordination between structurally segregated brain regions. Thus, studies using diffusion tensor imaging (DTI) and resting-state functional magnetic resonance imaging (rs-fMRI) provide additional insights into the neurological basis of the TS cognitive profile. While DTI allows researchers to examine the integrity of axonal pathways connecting different brain regions, functional connectivity analyses of rs-fMRI imaging data provide insight into the organization of large-scale brain networks supporting these processes. In older children, adolescents, and adults, individuals with TS exhibit distinct white matter fiber properties. Multiple studies have reported reduced fractional anisotropy (FA) in the superior longitudinal fasciculus (SLF), while others report more global reductions (Holzapfel et al. 2006; Yamagata et al. 2012; Villalon et al. 2013). In typically developing children, the SLF facilitates working memory, language, visuospatial attention, and numerical tasks (van Eimeren et al. 2010; Thiebaut de Schotten et al. 2011; Vestergaard et al. 2011; Urger et al. 2015), all domains affected in TS individuals. Using task-based fMRI, Bray and colleagues (Bray et al. 2013) were the first to demonstrate abnormal connectivity in parieto-occipital and parieto-temporal pathways in TS, which could explain deficits in visuospatial processing. Reduced functional connectivity at rest has been reported in the frontoparietal and dorsal attention networks in girls with TS, which may explain the increased prevalence of ADHD in the TS population (Green et al. 2018). Whole-brain reduction in functional connectivity strength was identified in the postcentral gyrus/intraparietal sulcus, angular gyrus, and cuneus and the right cerebellum in girls with TS (Xie et al. 2017). This study also showed a relationship between these connectivity deficits and cognitive domains such as working memory and visuospatial reasoning.

The above studies using DTI and rs-fMRI were conducted in older children, adolescents, and adults, leaving the question of when in development the observed differences in white matter microstructure and functional connectivity arise unanswered. Consequently, it is unclear whether observed differences are a direct consequence of hemizygosity of X-chromosome genes expressed during the prenatal and early postnatal period when axonal pathways and functional networks are first established, or occur later, when the dominant processes influencing connectivity are myelination and axon pruning. If these differences do arise later in childhood, they could reflect postnatal deficiency in gonadal steroid hormones (Rubin et al. 1981; Schulz and Sisk 2016; Klein et al. 2018). Most girls with TS in developed countries also undergo treatment with growth hormone to increase adult height (Blunden et al. 2018). Compared to typical children, they are more likely to take additional medications for a variety of issues (e.g. hypertension, middle ear disease, autoimmune thyroid disease, attention deficit disorder, and metabolic dysfunction) and to undergo surgery with general anesthesia (Davenport 2010), all with potential consequences for brain development.

The current study focuses on 1-year-old infants who have not yet been exposed to growth hormone or estrogen replacement therapy. Our main objective was to determine whether anatomical and functional connections between frontal and parietal/occipital cortices are altered in 1-year-old infants with TS. We addressed this objective through a cross-sectional case–control study of TS and typically developing (TD) infants with DTI and rs-fMRI scanning data. Based on the literature summarized above, we hypothesized that infants with TS would have aberrant diffusivity in the SLF and reduced functional connectivity between the precentral gyrus and regions involved in basic visual processing (calcarine cortex), SC (supramarginal gyrus and lingual cortex), and EF (supramarginal gyrus). We focused on these specific regions because they showed volume reductions in a prior study of the same cohort (Davenport et al. 2020). To our knowledge, this study is the first of its kind to utilize rs-fMRI and DTI together in TS to answer questions about brain structure and function at this developmental stage. Our results have the potential to show whether differences in white matter structural properties and functional connectivity observed in older children and adults with TS are present in infancy versus emerging at a later developmental stage. This has implications for our understanding of the neurodevelopmental processes in TS and lays the foundation for early intervention strategies and therapies for TS that could help ameliorate neurocognitive deficits in the disorder.

## Materials and methods

### Participants

Imaging data from 26 females with X monosomy (6 mosaic and 20 with complete X monosomy), 39 typically developing males, and 47 typically developing females were used in this study. All participants were approximately 1 year of age. Twenty-one individuals with TS were used in the functional connectivity portion of this study, with 47 TD females and 39 TD males. Usable DTI scans were available for 24 females with X monosomy, 31 TD males, and 36 TD females. TS participants were recruited through a large regional health system in the Southeastern United States, national support groups, and heath care providers from across the United States. Typically developing subjects represent a subset of the Early Brain Development Study (EBDS) cohort at the University of North Carolina at Chapel Hill (Knickmeyer et al. 2008, 2017; Gilmore et al. 2010). Exclusion criteria for both groups included substance abuse or major health problems in the mother during pregnancy, major psychiatric illness in either parent, extreme prematurity of the child, and any congenital abnormality in the subject not associated with TS. This study was approved by the Institutional Review Boards of the University of Chapel Hill (UNC) School of Medicine and Michigan State University. Written informed consent was obtained from a parent or legal guardian prior to the study.

### rs-fMRI and DTI acquisition and processing

Imaging data were acquired over a 10-year period on either a 3T Siemens Allegra head-only or a 3T Siemens Tim Trio scanner (Siemens Medical System, Inc., Erlangen, Germany) that replaced the Allegra part way through the study. There were 31 infants imaged on the Tim Trio and 81 on the Siemens Allegra. All infants were in a natural sleep state during imaging.

Functional imaging was performed using a T2-weighted echo planar imaging (EPI) sequence: TR = 2,000 ms, TE = 32 ms, 33 slices, and 4 mm isotropic resolution, while structural images were acquired using 3D magnetization–prepared rapid acquisition with gradient echo (MPRAGE) sequence, which is a T1-weighted imaging technique. The sequence is as follows: TR = 1,820 ms, TE 4.38 ms, and 1 mm isotropic resolution. These methods have been described previously (Shi et al. 2018).

A 6-direction protocol with the following parameters was used to collect DTI data on the Allegra during the initial years of the study (81 infants total): repetition time (TR)/echo time (TE) = 5,200/73 ms, slice thickness = 2 mm, and in-plane resolution = 2 × 2 mm^2^, with a total of 45 slices in 6 unique directions using the *b* value of 1,000 s/mm^2^ and 1 baseline image (*b* value = 0) per sequence. In total, 35 diffusion-weighted images (DWIs) were generated per subject by repeating the sequence five times to improve the signal-to-noise ratio. Later imaging on the Allegra used 42 directions of diffusion sensitization with a *b* value of 1,000 s/mm^2^ in addition to seven baseline images, which generated a total of 49 DWIs. The parameters for the 42-direction data were as follows: TR/TE/flip angle = 7,680/82/90°, slice thickness = 2 mm, and in-plane resolution = 2 × 2 mm^2^, with a total of 60 to 72 slices. The remainder of subjects were scanned on the Tim Trio following the same parameters as the 42-direction Allegra protocol. These methods are described in Girault et al. (2019). Data scrubbing (Power et al. 2012) was implemented as an additional motion correction step. Volumes with frame-wise displacement (FD) > 0.3 mm were removed (i.e. “scrubbed”) from the data; if fewer than three volumes remained between the scrubbed volumes, then these volumes were also removed (Power et al. 2012).

### Image analysis (rs-fMRI)

Functional data were preprocessed using the FMRIB (Functional MRI of the Brain) Software Library (FSL; version 6.0; Smith et al. 2004) and Analysis of Functional NeuroImages (AFNI; Cox 1996). Steps included discarding the first 3 volumes (6 s), slice-timing correction, rigid-body motion correction, bandpass filtering (0.01 to 0.08 Hz), and regression of white matter, CSF, the 6 motion parameters, and their derivative, quadratic terms. The nuisance signals were bandpass-filtered before regression to match the frequency of the blood oxygen level–dependent signal. A single global signal regression step was performed after the scrubbing to eliminate global signal re-introduced by scrubbing. Data scrubbing was implemented with scrubbing criteria at global signal changes > 0.5% and framewise displacement > 0.3 mm (Power et al. 2011) with continuous datapoints no less than 3. Participants with less than 3 min (90 datapoints) of functional data after scrubbing were excluded. Five subjects were excluded (two with TS). Then, to make the same length of data, all infants’ functional data were truncated to 90 volumes (3 min). Finally, the images were spatially smoothed with Gaussian kernel (full width at half maximum = 6 mm).

After performing a rigid-body registration between the functional data and T1-weighted structural images of the same subject, a nonlinear registration (FMRIB nonlinear registration (fNIRT) in FSL) was done between individual T1-weighted images and infant age–specific automated anatomical labeling atlas (AAL template images (Shi et al. 2011). The combined transformation field was used to warp the preprocessed rs-fMRI functional images to the neonate template (Shi et al. 2018). The structural image skull-stripping was done using a template-based method. After performing a rigid-body registration between the functional data and T1-weighted structural images of the same subject, a nonlinear registration (fNIRT in FLS) was done between individual T1-weighted images and an infant-specific AAL template (Shi et al. 2011) that includes 90 regions of interest. The combined transformation field was used to warp the preprocessed rs-fMRI functional images to the group template (Shi et al. 2018).

For the current analysis, we extracted the average blood-oxygenation-dependent-level (BOLD) time course from the right precentral gyrus, right and left calcarine cortex, right and left lingual cortex, and right supramarginal cortex (regions that showed reduced volumes in infants with TS as per Davenport et al. (2020). Correlations between the right precentral gyrus and the other five regions were then calculated for each subject. The correlations were then Fisher-*Z*-transformed for statistical analysis.

### Statistical analyses (rs-fMRI)

All statistical analyses were performed using R statistical software 4.0.5 (R Core Team 2021) using base functions.

To evaluate group differences in demographic and medical history variables, we conducted two-sided Fisher’s exact tests for categorical variables and two-sided Kruskal–Wallis H-tests for continuous variables with a Dunn test used for post hoc comparisons.

A one-way analysis of covariance (ANCOVA) was used to test differences in functional connectivity between the three groups (TS females, XX females, XY males) with post-hoc false discovery rate (FDR)-corrected pairwise comparisons. Five outcome variables were examined: (i) connectivity between the right precentral gyrus and the right calcarine cortex, (ii) connectivity between the right precentral gyrus and the left calcarine cortex, (iii) connectivity between the right precentral gyrus and the right lingual cortex, (iv) connectivity between the right precentral gyrus and the left lingual cortex, and (v) connectivity between the right precentral gyrus and the right supramarginal cortex. To reduce the variance, covariates were chosen that are variables previously associated with imaging outcomes: scanner type, birth weight, framewise displacement used as a motion correction variable, and gestational age at MRI (Blanchett et al. 2023). Analyses were performed with and without global signal regression.

### Exploratory analysis (rs-fMRI)

In addition to our primary analysis, which focused on the five outcome variables described above, we conducted an exploratory whole-brain analysis for each of the six brain regions that showed volume reduction in our prior study of infants with TS: right precentral gyrus, right calcarine cortex, left calcarine cortex, right lingual cortex, left lingual cortex, and right supramarginal cortex. Correlations between the average BOLD time course for each region and all 90 regions in the AAL atlas were calculated for each subject. Group comparisons were conducted via one-way ANCOVA as previously described, incorporating the same covariates. As before, analyses were performed with and without global signal regression.

### Image analysis (DTI)

All image analysis steps for DTI are described in Girault et al. (2019) and represent an infant-specific pipeline adapted from the general UNC-Utah NA-MIC DTI pipeline (Verde et al. 2014). Quality control (QC) is an automated protocol using DTIPrep (Oguz et al. 2014). DTIPrep detects and excludes images corrupted by artifacts in DWI sequences and corrects for eddy currents (Oguz et al. 2014). Images with large motion artifacts and aberrant gradients are excluded. Weighted least squares fitting was used to estimate the diffusion tensors. Additional expert-guided QC was performed using 3D Slicer. Skull stripping was performed as specified in Verde et al. (2014). A DTI atlas derived from 1-year-olds was used to map the images. Successful registration was confirmed by visually comparing the warped DTI images and the atlas. Atlas fibers were then mapped onto the individual subject space, and DTIAtlasFiberAnalyzer was used to extract the FA, mean diffusivity (MD), radial diffusivity (RD), and axial diffusivity (AD) profiles in each tract. Functional Analysis of Diffusion Tensor Tract Statistics (FADTTS) (Gao et al. 2009; Zhu et al. 2011) was applied using FADTTSter (Noel et al. 2017), which assessed the quality of the extracted fiber profiles via a correlation analysis against the FA. Profiles with a correlation value above 0.7 were considered well mapped to the atlas space; profiles that did not meet this criterion were excluded (Stephens et al. 2020).

### Statistical analyses (DTI)

As described in Jha et al. (2018), FADTTS, or functional analysis of diffusion tensor tract statistics (Zhu et al. 2011), was used to test group differences in FA, AD, and RD along the left SLF white matter tract. Insufficient data were available to test for differences in the right SLF. MD was not included as it is a general measure of diffusivity, making it challenging to interpret biologically, and is calculated using AD and RD creating issues with collinearity. FADTTS yields a global test statistic and local test statistics along the white matter fiber tracts. We included DTI protocol, ICV, postnatal age at MRI, gestational age at birth, and two motion measures as covariates. The first motion measure was calculated by taking the sum of volumes excluded due to artifacts along with the number of excluded volumes due to residual motion. The second measurement is the number of volumes exceeding major motion as determined by rotational motion > 1 degree and translational motion > 2 mm. DTI protocol and scanner type were controlled for by creating three dummy variables within the FADTTS analysis indicating use of the Allegra or Trio and the number of directions: A06 or A42 in the case of the Allegra, or just T42 in the case of the Trio.

Within each tract, local *P*-values are corrected for multiple comparisons with FDR. The local *P*-values are then merged with the test statistics onto the corresponding fiber locations for visualization.

### Exploratory analyses (DTI)

The processes for DTI image and statistical analyses are the same as those described for the SLF. A total of 52 tracts were generated, which include, bilaterally, the arcuate fasciculus (frontoparietal, frontotemporal, and temporoparietal segments), anterior portion of the cingulum, posterior cingulum adjoining the hippocampus, corticofugal tracts (motor, parietal, prefrontal, premotor segments), corticoreticular tract, corticospinal tract, corticothalamic tracts (motor, parietal, prefrontal, premotor, superior segments), fornix, fronto-occipital fasciculus, inferior longitudinal fasciculus, optic tract, optic radiation, and uncinate fasciculus, as well as segments of the corpus callosum (body, genu, motor, parietal, premotor, rostrum, splenium, and tapetum). However, only 45 tracts passed quality control and had an *n* greater than or equal to 20 for each group. Tracts excluded from the exploratory statistical analyses include: the right temporoparietal portion of the arcuate fasciculus, left corticospinal tract, left optic tract, right premotor and prefrontal corticofugal tracts, and left premotor and prefrontal corticofugal tracts.

## Results

### Participants

Descriptive statistics for the demographic and medical history variables of the participants in this study are shown in Table 1. Significant differences were found between the three groups in gestational age at birth, birthweight, scanner type, and maternal ethnicity. On average, children with TS were born earlier than control participants and had the lowest birthweight. The children with TS were more likely to be scanned on the Tim Trio than the Allegra and were more likely to be White.

**Table 1:** Demographics and medical history for 1-year-old infants with TS and their typically developing male and female counterparts.

**Variable**	**Turner syndrome**		**Female control**		**Male control**		***P***
	***N***	**Mean (SD) range**	***N***	**Mean (SD) range**	***N***	**Mean (SD) range**	
Gestational age at birth (days)	26	268 (11) 246 to 286	47	277 (9) 259 to 295	39	274 (10) 241 to 289	0.006
Birth weight (grams)	26	2,803 (421) 2155 to 3925	47	3,380 (428) 2340 to 4414	39	3,386 (469) 2375 to 4562	<.0001
Age at MRI (days)	26	389 (16) 359 to 419	47	382 (22) 339 to 439	39	382 (18) 343 to 422	0.357
Maternal age (years)	25	29 (6) 21 to 42	47	30 (5) 20 to 40	39	30 (4) 20 to 41	0.531
Paternal age (years)	25	32 (7) 23 to 50	47	32 (6) 21 to 50	37	32 (4) 22 to 39	0.842
Maternal education (years)	25	15 (3) 6 to 20	47	16 (3) 9 to 23	39	16 (3) 10 to 22	0.235
Paternal education (years)	24	14 (3) 3 to 23	46	16 (3) 8 to 22	39	16 (3) 9 to 22	0.093
Total household income (dollars)	24	76,095 (69,420) 0 to 330,000	45	68,280 (46,264) 0 to 205,000	37	73,562 (47,217) 0 to 195,000	0.913
	*N*	%	*N*	%	*N*	%	
Maternal ethnicity							0.0181
White	23	92.0%	32	68.1%	35	89.7%	
Black	2	8.0%	13	27.7%	2	5.1%	
Asian	0	0.0%	2	4.3%	2	5.1%	
Paternal ethnicity							0.056
White	23	92.0%	32	68.1%	33	86.8%	
Black	2	8.0%	13	27.7%	3	7.9%	
Asian	0	0.0%	2	4.3%	2	5.3%	
Smoking							0.843
Yes	1	3.8%	3	6.4%	1	2.6%	
No	25	96.2%	44	93.6%	38	97.4%	
Scanner							0.0185
Trio	13	50.0%	11	23.4%	7	17.9%	
Allegra	13	50.0%	36	76.6%	32	82.1%	
Allegra directionality							
A06	5	45%	9	35%	9	35%	0.834
A42	6	55%	17	65%	17	65%	

### Connectivity measures

The one-way ANOVAs were in the expected direction apart from the right supramarginal gyrus in which connectivity in TS is higher than in the other groups. Our hypothesis was that reduced connectivity would be seen in the TS group. No statistically significant group differences were found after FDR correction (Table 2). Effect sizes as calculated by eta squared were 0.07 (medium; right calcarine cortex), 0.07 (medium; left calcarine cortex), 0.085 (medium; right lingual cortex), 0.063 (medium; left lingual cortex), and low at 0.033 for the right supramarginal gyrus. The right precentral gyrus to the right lingual cortex had an uncorrected *P*-value below 0.05 and an FDR-corrected *P*-value near 0.10. We proceeded with a pairwise comparison for this phenotype and found significant differences between XX females and X0 females (*P* = 0.03), though this was not significant after FDR correction (*P* = 0.09) (Table 3). X0 females showed stronger anticorrelations than XX females. ANCOVAs performed on data without global signal regression also showed no statistically significant results after FDR adjustment (Supplementary Table 1). *P*-values were generally larger for the analysis without signal regression with the results for the right lingual cortex having a value of 0.051, uncorrected. Effect sizes as calculated by eta squared were 0.054 (medium; right calcarine cortex), 0.051 (medium; left calcarine cortex), 0.078 (medium; right lingual cortex), 0.028 (low; left lingual cortex), and 0.013 (low; right supramarginal gyrus).

**Table 2:** One-way ANCOVA results for resting-state functional connectivity between the right precentral gyrus and five occipital-parietal regions showing reduced volumes in infants with TS using global signal regression.

**Region**	**LS (SE) TS**	**LS (SE) female**	**LS (SE) male**	**Uncorrected *P*-value**	**FDR corrected *P*-value**
Right calcarine cortex	−0.235 (0.09)	0.059 (0.073)	−0.014 (0.077)	0.065	0.108
Left calcarine cortex	−0.277 (0.087)	0.013 (0.07)	−0.085 (0.074)	0.059	0.108
Right lingual cortex	−0.203 (0.079)	−0.089 (0.065)	0.011 (0.069)	0.035	0.108
Left lingual cortex	−0.205 (0.077)	0.029 (0.063)	−0.071 (0.066)	0.093	0.116
Right supramarginal gyrus	0.286 (0.098)	0.17 (0.079)	0.073 (0.084)	0.297	0.297

**Table 3:** Pairwise comparison of the resting-state functional connectivity between the right precentral gyrus and right lingual cortex. Please note that this post hoc analysis was run because the ANCOVA for this phenotype had an uncorrected *P*-value below 0.05 and an FDR-corrected *P*-value near 0.10; the original ANCOVA did not survive FDR.

**Region**	**Comparison**	**Estimate**	**SE**	***P*-value**
Right lingual cortex				
	XX-XY	0.078	0.086	1.00
	XX-X0	0.291	0.11	0.03
	XY-X0	0.213	0.109	0.16

Results of the exploratory analyses can be found in Supplementary Tables 2 and 3, which show the ANCOVA results and post hoc pairwise comparisons, respectively. The only two measures to survive FDR correction were connectivity between the right supramarginal gyrus and the left insula (with global signal regression) and between the right supramarginal gyrus and the right putamen (with global signal regression) (see Supplementary Table 4 for group LS Means). However, it is interesting to note that several regions exhibited uncorrected *P*-values less than 0.05 in 6 or more of the 12 exploratory analyses conducted. This includes the left posterior cingulate gyrus, the left postcentral gyrus, the right putamen, and the right pallidum.

### DTI measures

In our primary analysis, which focused on the left SLF, we did not observe statistically significant differences between individuals with TS and male or female controls (Table 4). The TS group generally had lower FA than female and male controls except at more anterior areas of the brain. The TS group also had lower AD than female controls and lower RD than male controls. Direction of effect varied substantially across the tract when comparing TS girls to male controls for AD and when comparing RD values in TS girls to control males. In all cases, beta values approached zero. The right SLF was not analyzed due to the previously described cutoff for subject numbers after quality control. With regard to motion as a potential factor in our results, the left SLF showed statistical significance in the first and second motion variable in comparisons involving XX females. Motion variable 1 was statistically significant in AD, while the second motion variable was statistically significant in AD (control female to control male comparison) and RD (control female to TS comparison).

**Table 4:** Paired comparisons between the three groups for white matter microstructure of the left superior longitudinal fasciculus. AD, RD, and FA are represented in the post hoc analysis and show no statistical significance.

			**FDR-corrected *P*-values**		
**Left SLF**	**Uncorrected global *P*-value**	**FDR-corrected global *P*-value**	*AD*	*RD*	*FA*
Female control, Male control	0.099	0.171	0.068	0.31	0.654
Female control, Turner syndrome	0.585	0.621	0.427	0.515	0.296
Male control, Turner syndrome	0.092	0.165	0.212	0.077	0.205

### Exploratory analyses

Of the 46 additional white matter tracts examined, we found significant differences between TS participants and controls in nine white matter tracts (Table 5): the frontotemporal segment of the right arcuate fasciculus, the right posterior cingulum adjoining the hippocampus, the motor and tapetum segments of the corpus collosum, the left motor segment of the corticofugal tract, the premotor segment of the right corticothalamic tract, the left inferior fronto-occipital fasciculus (IFOF), the left inferior longitudinal fasciculus, and the right optic tract. Global omnibus and FDR *P*-values and *P*-values for AD, RD, and FA for all 46 tracts that passed quality control are found in Supplementary Table 5.

**Table 5:** Fasciculi with statistically significant FDR-corrected global *P*-values and post hoc FDR-corrected *P*-values for individual diffusivity metrics. AD indicates axial diffusivity, RD indicates radial diffusivity, and FA indicates fractional anisotropy.

			Post hoc FDR-corrected *P*-values		
	Global *P*-value	Global FDR-corrected *P*-value	AD	RD	FA
**Arcuate fasciculus, right, frontotemporal**					
Female control, Turner syndrome	0.007	0.033	0.009	0.224	0.078
Male control, Turner syndrome	0.387	0.455	0.329	0.725	0.319
Female control, Male control	0.026	0.076	0.062	0.013	0.255
**Cingulum adjoining the hippocampus, right**					
Female control, Turner syndrome	<0.0001	<0.00092	0.001	0.002	0.197
Male control, Turner syndrome	0.466	0.522	0.608	0.87	0.899
Female control, Male control	<0.0001	<0.00092	0.004	<0.0001	0.526
**Corpus callosum, motor**					
Female control, Turner syndrome	0.007	0.033	0.027	0.426	0.261
Male control, Turner syndrome	0.607	0.644	0.419	0.584	0.477
Female control, Male control	0.013	0.054	0.099	0.863	0.641
**Corpus callosum, tapetum**					
Female control, Turner syndrome	0.004	0.022	0.042	0.241	0.011
Male control, Turner syndrome	0.051	0.1104	0.178	0.505	0.09
Female control, Male control	0.312	0.403	0.84	0.941	0.104
**Corticofugal, left, motor**					
Female control, Turner syndrome	<0.0001	<0.00092	<0.0001	<0.0001	0.021
Male control, Turner syndrome	<0.0001	<0.00092	0.004	0.087	<0.0001
Female control, Male control	<0.0001	<0.00092	<0.0001	<0.0001	0.654
**Corticothalamic, right, premotor**					
Female control, Turner syndrome	0.003	0.018	0.005	0.311	0.129
Male control, Turner syndrome	0.452	0.515	0.319	0.574	0.626
Female control, Male control	<0.0001	<0.00092	<0.0001	0.338	0.008
**Inferior fronto-occipital fasciculus, left**					
Female control, Turner syndrome	0.032	0.083	0.088	0.14	0.046
Male control, Turner syndrome	0.001	0.007	0.003	0.066	0.012
Female control, Male control	0.029	0.080	0.075	0.378	0.077
**Inferior longitudinal fasciculus, left**					
Female control, Turner syndrome	0.001	0.007	0.201	0.044	0.039
Male control, Turner syndrome	0.028	0.078	0.339	0.247	0.073
Female control, Male control	0.017	0.065	0.045	0.801	0.313
**Optic tract, right**					
Female control, Turner syndrome	0.035	0.087	0.406	0.211	0.388
Male control, Turner syndrome	0.008	0.035	0.145	0.06	0.007
Female control, Male control	0.024	0.073	0.098	0.084	0.018

When considering the motion covariates used in the DTI exploratory analyses, all fibers but the left IFOF showed statistical significance in one or more diffusivity measures for both the first and second motion variables. The right optic tract displayed statistical significance in the female-Turner and male-Turner comparison for all diffusivity measures for the first motion variable, and statistical significance in all three diffusivity measures in the male-Turner comparison in the second motion variable. Overall, 20% of diffusivity measures showed statistically significant effects from both the first and second motion variables.

AD was statistically significant in the right frontotemporal segment of the arcuate fasciculus (*P* = 0.009 female control vs TS), the right posterior cingulum adjoining the hippocampus (*P* = 0.004 female control vs male control, *P* = 0.001 female control vs TS), the motor segment of the corpus callosum (*P* = 0.024, female control vs TS), the tapetum segment of the corpus callosum (*P* = 0.042, female control vs TS), and the left motor segment of the corticofugal tract (*P* = <0.0001 in both female control vs male control and female control vs TS, *P* = 0.004 for male control vs TS). Additionally, the premotor portion of the right corticothalamic tract (*P* < 0.0001 female control vs male control and *P* = 0.005 for female control vs TS) and the left IFOF (*P* = 0.003 male control vs TS) all present with statistically significant FDR-corrected *P* values.

RD showed statistical significance in the right posterior cingulum adjoining the hippocampus (*P* = 0.002 female control vs TS, and *P* < 0.0001 female vs male controls), the left motor section of the corticofugal tract (*P* = 0.024 female vs male control; *P* < 0.0001 female control vs TS), and the left inferior longitudinal fasciculus (*P* = 0.044 female control vs TS).

Finally, FA was statistically significant in the tapetum of the corpus collosum (*P* = 0.011, female control vs TS), the left motor segment of the corticofugal tract (*P* = 0.021, female control vs TS; *P* < 0.0001, male control vs TS), the left IFO (*P* = 0.046, female control vs TS; 0.012 male control vs TS), the left inferior longitudinal fasciculus (*P* = 0.039, female control vs TS), and the right optic tract (*P* = 0.007, male control vs TS).

**Fig. 1:**
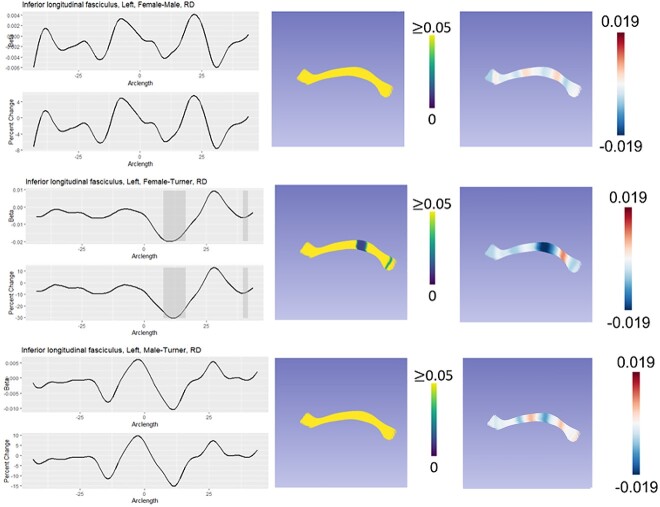
Model of DTI results for the left inferior longitudinal fasciculus. In each panel, the leftmost graph shows the beta value over the arclength of the fasciculus. Areas of local statistical significance are highlighted in gray. Below that is the % change between groups. The middlemost image in each panel shows an overlay of the local *P*-values on the fasciculus, with regions of statistical significance showing a color other than yellow. Finally, the rightmost panel shows the beta values from the specific comparison with the model of the tract. Here, the inferior longitudinal fasciculus is an example of a PAR pattern.

**Fig. 2:**
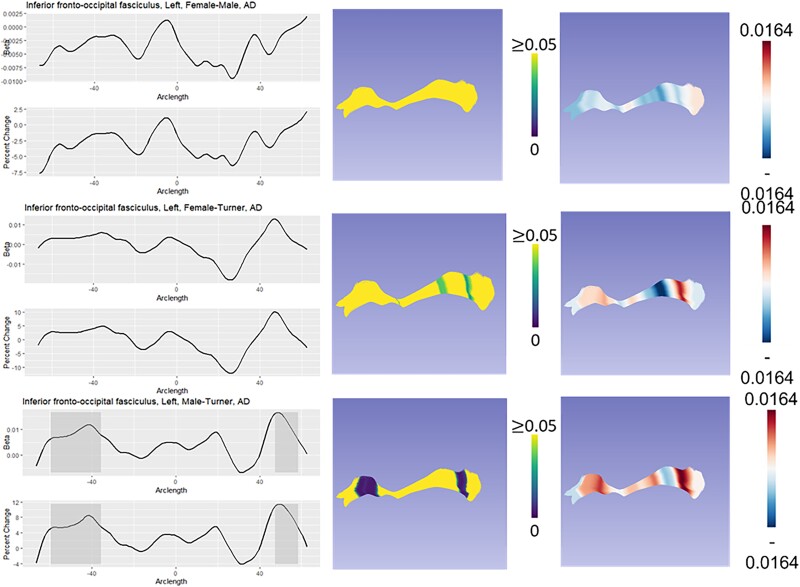
Model of DTI results for the left IFOF. In each panel, the leftmost graph shows the beta value over the arclength of the fasciculus. Areas of local statistical significance are highlighted in gray. Below that is the % change between groups. The middlemost image in each panel shows an overlay of the local *P*-values on the fasciculus, with regions of statistical significance showing a color other than yellow. Finally, the rightmost panel shows the beta values from the specific comparison with the model of the tract. Here, the anterior portion of the IFOF is an example of a sex difference.

**Fig. 3:**
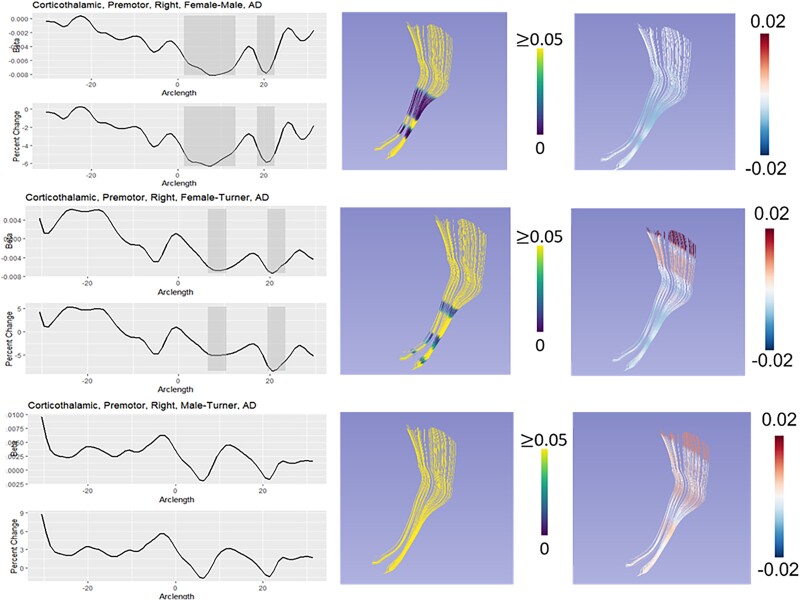
Model of DTI results for the right premotor portion of the corticothalamic tract. In each panel, the leftmost graph shows the beta value over the arclength of the fasciculus. Areas of local statistical significance are highlighted in gray. Below that is the percent change between groups. The middlemost image in each panel shows an overlay of the local *P*-values on the fasciculus, with regions of statistical significance showing a color other than yellow. Finally, the rightmost panel shows the beta values from the specific comparison with the model of the tract. Here, the corticothalamic tract is an example of a masculinization.

We classified group differences into six categories based on both global and local test statistics: XX > X0 = XY, X0 = XY > XX, X0 > XY = XX, XX = XY > X0, X0 = XX > XY, and XY > X0 = XX. We refer to both XX > X0 = XY and X0 = XY > XX as “masculinization” patterns because the TS females are similar to TD males but differ from TD females. We refer to both X0 > XY = XX and XX = XY > X0 as “PAR” patterns because they suggest a role for genes in the pseudoautosomal regions of the sex chromosomes. Finally, we refer to both X0 = XX > XY and XY > X0 = X as “sex difference” patterns because phenotypic females (TS and TD) differ from TD males. The primary pattern for each diffusivity measure and each tract that showed significant group difference in post hoc analyses is shown in Table 6. Figures 1–3 illustrate how these three different patterns manifest in the right premotor portion of the corticothalamic tract, the left inferior longitudinal fasciculus, and the left inferior fronto-occipital fasciculus. All other fasciculi showing significant group differences can be seen in Supplementary Figs. 1–12.

**Table 6:** **Chromosomal hierarchies for each statistically significant tract shown for axial diffusivity, radial diffusivity, and fractional anisotropy.** XX indicates typically developing female, X0 indicates TS female, and XY indicates typically developing male. Classifications in this table represent the dominant pattern we observed. However, examination of local tract statistics often revealed regional complexities, which we describe in more detail in the discussion section.

**Fasciculus**	**Axial Diffusivity**	**Radial Diffusivity**	**Fractional Anisotropy**
Arcuate, right, frontotemporal	XX > X0 = XY		
Cingulum adjoining hippocampus, right	XX > X0 = XY	XX > X0 = XY	
Corpus callosum, motor	X0 = XY > XX		
Corpus callosum, tapetum	XX = XY > X0		XX = XY > X0
Corticofugal, left, motor	XX > X0 = XY	XX > X0 = XY	X0 > XY = XX
Corticothalamic, right, premotor	XX > X0 = XY		
Inferior fronto-occipital fasciculus, left	XX = X0 > XY		X0 = XX > XY
Inferior longitudinal fasciculus, left		XX = XY > X0	X0 > XX = XY
Optic tract, right			X0 = XY > XX

## Discussion

The current work is, to our knowledge, the first to use rs-fMRI and DTI to study infants with TS. Overall, our study suggests a unique developmental pattern in TS in which functional connectivity differences and differences in white matter microstructure arise at varying times in the life span. Our primary analyses indicate that diffusivity differences in the SLF are not present at 1 year of age and presumably emerge during early childhood, as they have been reported in older children and adults. Similarly, significant differences in functional connectivity between frontal and parietal/occipital regions were not detected in the current study but are evident later in life. Our exploratory rs-fMRI analyses revealed that infants with TS may have altered connectivity between the right supramarginal gyrus and the left insula and right putamen. Our exploratory DTI analyses revealed nine fasciculi that differed between TD and TS infants.

Our original hypothesis that infants with TS would have reduced functional connectivity between the precentral gyrus and our five selected regions came from previous work showing volumetric differences in the brains of the same cohort of individuals (Davenport et al. 2020). The five selected regions also contribute to cognitive functions that are often disrupted in TS, supporting visual processing (left and right calcarine cortex), EF (supramarginal gyrus), and SC (left and right lingual cortex). It is intriguing that there are such pronounced volumetric differences in this cohort of infants, but no significant differences were observed in functional connectivity, especially as disrupted connectivity between frontal and parietal regions has been reported in older individuals with TS (Frye et al. 2010; Kamali et al. 2014; Rizio and Diaz 2016; Koyama and Domen 2017). That being said, effect sizes were medium for all comparisons except functional connectivity between the right precentral gyrus and the right supramarginal gyrus. Furthermore, one connectivity pair had uncorrected *P*-values below 0.05 and FDR-corrected *P*-values near 0.10. In this case, X0 females showed anticorrelations that were larger than those observed in the other groups. Our previous studies have shown the emergence of anticorrelations during the first year of life (Gao et al. 2013; Salzwedel et al. 2019), especially between the default-mode network and the dorsal attention network (Gao et al. 2013), suggesting the gradual online of adult-like “competition” between internal- and external-driven domains of brain functioning (Fox et al. 2005; Gao and Lin 2012) during infancy. However, anticorrelations between motor and visual areas were not robustly observed in typically developing infants (Gao et al. 2015a; Gao et al. 2015b). The findings in this study that infants with TS showing stronger anticorrelations between motor-related precentral gyrus and visual-related calcarine and lingual areas than control groups may indicate an aberrant growth of anticorrelations between the two primary sensory domains in TS, which may elicit stronger competition/suppression between the two domains and underlie the reported visual spatial deficits and attentional-related difficulties observed later in life in children with TS. A larger study is needed to confirm if these differences can be independently reproduced and related to later behavioral outcomes. If so, it might suggest that earlier connectivity alterations between the sensorimotor network and visual networks in TS might contribute to later visuospatial and motor-perceptual challenges, with perhaps pre-emptive prophylactic interventions being considered (e.g. parent engagement around infant movement and sensory experiences). Our exploratory rs-fMRI analyses suggests that infants with TS may have altered connectivity between the right supramarginal gyrus and the left insula and right putamen. The TS infants showed greater connectivity than the typically developing female and male infants, both of which showed minimal connectivity between these particular regions. Alterations in insula volume and cortical thickness have been reported in older individuals with TS (Marzelli et al. 2011; Lepage et al. 2014), and one study has reported increased gray matter volume in the putamen (Zhao et al. 2013). The insula is involved in a variety of functions including SC and sensorimotor processing, but functional connectivity between the dorsal anterior insula and parietal areas may be particularly important for cognitive control (Uddin et al. 2017) and SC (Zhao et al. 2021). The putamen is well known for its role in in regulation of motor activity and movement but may also play a role in visual processing (Kunimatsu et al. 2019; Vicente et al. 2012), suggesting that increased functional connectivity between supramarginal gyrus and right putamen in TS may be a compensatory strategy to address volume loss in visuo-spatial areas.

For the white matter structural properties, we hypothesized that there would be diffusivity differences between TS infants and TD infants in the SLF, a tract that connects frontal and parietal brain regions and is involved in EF, attention, and working memory (Frye et al. 2010; Kamali et al. 2014; Madhavan et al. 2014; Rizio and Diaz 2016; Koyama and Domen 2017). This hypothesis was based on the extant literature in adults (Holzapfel et al. 2006), peripubertal girls (Villalon et al. 2013), and prepubescent girls with TS (Yamagata et al. 2012). However, we did not observe significant differences between groups in the current study. Integrating our results with the existing literature suggests the SLF develops normally in TS until at least 1 year of age but deviates during the toddler and preschool years. We were not able to study the right SLF, and future studies should examine this pathway via similar strategies to examine for any aberrant development in very young females with TS.

Null findings for fronto-parietal connections, assessed with both DTI and rs-fMRI, parallel findings on early cognitive development in TS. Infants with TS show a relatively normal cognitive profile at 12 months of age with a potentially slower rate of progression in visual reception and fine motor skills from 12 to 24 months (Pretzel et al. 2020). If differences in fronto-parietal connections arise after 12 months of age, there may be an opportunity to prevent those changes through early intervention as noted above, thereby preventing or ameliorating some of the cognitive impairments observed in school-aged children with TS. Historically, diagnosis of TS was made when a female failed to go through puberty or fell two or more standard deviations below the mean height for their age group, resulting in relatively late diagnosis and reduced intervention opportunities (Sävendahl and Davenport 2000; Moreno-García et al. 2005). However, characteristics such as lymphedema in infancy, presence of cardiac abnormalities, which occur in approximately 50% of the TS population (Sävendahl and Davenport 2000; Bondy 2008; Eckhauser et al. 2015), and the advancement of prenatal diagnostics such as genetic amniocentesis, sonograms, and circulating cell-free DNA have contributed to earlier diagnoses. Recent clinical practice guidelines for TS encourage annual developmental and behavioral screenings for TS and recommend academic adjustments to accommodate learning and performance issues (Gravholt et al. 2017). They also suggest that evidence-based interventions for cognitive or psychosocial problems in other populations be adapted to meet the needs of those with TS across the lifespan. The possible benefits of administering early interventions in a proactive manner, before cognitive deficits emerge, is not addressed. Early interventions clearly improve cognitive and psychosocial outcomes for individuals with ADHD (McGoey et al. 2002; Jones et al. 2007; Re et al. 2015) and ASDs (Eapen et al. 2013; Zwaigenbaum et al. 2015), conditions that share key characteristics with TS. Especially relevant to the current study, it has been shown that white matter diffusivity measurements improve with therapy in toddlers and preschoolers with ASD (Saaybi et al. 2019), and both functional and structural connectivity have been proposed as potential biomarkers for monitoring early interventions (Swanson and Hazlett 2019).

This leads directly into our exploratory DTI analysis, which revealed that some white matter tracts already display statistically significant differences at 1 year of age between the three groups. The majority of these exploratory findings followed a pattern in which TS infants differed from XX females but were similar to XY males. In other words, they suggest masculinized development of these tracts in TS, which could contribute to the increased risk for male-biased neurodevelopmental disorders. In tract-wide comparisons, this pattern was observed for specific diffusivity metrics in the right arcuate fasciculus (AD), right posterior cingulum (AD & RD), the motor segment of the corpus callosum (AD), the left corticofugal motor tract (AD, RD), and the right premotor corticothalamic tract (AD). Examination of local statistics revealed that this pattern was also present in portions of the left corticofugal motor tract (FA), left IFOF (AD & FA), left ILF (RD), and right optic tract (FA). Two possible explanations exist for this pattern, one being the relative lack of postnatal estrogen exposure in TD males and TS females. Another possibility is that these tracts are influenced by genes that lie outside the pseudoautosomal region but escape X-inactivation. Like TD males, TS girls are hemizygous for these genes. Interestingly, this pattern was generally observed in relation to AD and AD was generally lower in TS females and XY males. AD is known to decrease sharply across the first year of life (Stephens et al. 2020). Thus, one could interpret high AD as delayed maturation and low AD as accelerated maturation. Alternatively, lower AD in infants with TS may reflect axonal damage (Winklewski et al. 2018), but this seems an unlikely explanation for the lower AD observed in XY males as compared to XX females.

The second most common pattern we observed was one in which TS infants differed from both XX females and XY males, who were similar to each other. In tract-wide comparisons, this pattern was observed for the tapetum ([AD & FA], left ILF [RD, FA]), left corticofugal motor tract (FA), and left IFOF (FA). Examination of local statistics revealed that this pattern was also present for AD in a posterior area of the left IFOF. This pattern suggests a role for dosage-sensitive genes in pseudoautosomal regions of the sex chromosomes, as TD males and females have two copies and TS females have one. However, this pattern could also reflect the influence of clinical characteristics that are more common in infants with TS such as congenital heart disease (CHD) and exposure to general anesthesia during surgeries to correct CHD, kidney abnormalities, or frequent ear infections. Interestingly, this pattern was seen most frequently for FA with TS females having higher FA than TD males or females. FA increases over development such that high FA can be interpreted as faster maturation and low FA as slower maturation. FA does not capture a single biological process, but reflects increasing myelination and axonal integrity (Hüppi et al. 1998).

The least frequently observed pattern was one in which TS infants were similar to XX females and both differed from XX males. We refer to this as the “sex difference” pattern, and it was observed in several regions of the left IFOF (FA) and the right optic tract (FA). This could indicate a role for testosterone in the development of these tracts. Testosterone is significantly higher in XY males as compared to XX females during mid-gestation and in the first 6 months of postnatal life (Forest et al. 1973; Reyes et al. 1974; Shigeo et al. 1977; Tapanainen et al. 1981; Andersson et al. 1998; Kuiri-Hänninen et al. 2011). Testosterone levels in TS are expected to be similar to XX females during gestation and even lower than XX females postnatally. It could also indicate a role for Y chromosome material in the development of these tracts. The relative lack of sex differences is in keeping with prior literature on the development of white matter integrity in infancy (Gilmore et al. 2018). In subsequent paragraphs, we discuss the pattern of results for each tract in more detail and discuss the potential functional and clinical implications of the patterns we observed.

The right frontotemporal segment of the arcuate fasciculus connects the middle and inferior temporal gyri with the precentral gyrus and posterior portion of the inferior and middle frontal gyri in the right hemisphere. This tract is involved in visuospatial processing and some aspects of language processing, especially prosody. Damage or underdevelopment of the right arcuate fasciculus is also associated with poorer ability in facial expression–based Theory of Mind (Nakajima et al. 2018), a subdomain of SC in which girls and women with TS are often deficient. We observed a pattern in which XX females had greater AD than females with TS, who were similar to XY males, which suggests that both TS females and XY males have more mature tracts than XX females. This seems slightly paradoxical given that this tract is involved in cognitive functions that are often disrupted in TS. Perhaps the development of this tract slows after 1 year of age in TS females, or it may asymptote at a lower level than XX females.

The right cingulum originates in the orbital frontal cortices, travels along the dorsal surface of the corpus callosum, then down the temporal lobe to the entorhinal cortex. In addition to linking frontal, parietal, and medial temporal sites, it also connects subcortical nuclei to the cingulate gyrus. We observed masculinization of both AD and RD in the posterior portion of the cingulum adjoining the hippocampus. Like AD, RD decreases over development. While changes in AD are thought to reflect increased numbers of fibers and/or axonal caliber, increased RD is thought to reflect myelination (Winklewski et al. 2018). As with the right frontotemporal segment of the arcuate fasciculus, our results suggest more rapid development of this tract in both XY males and TS females. The right cingulum is known to be involved in general cognition and has been linked to a myriad of psychiatric disorders including ASD and ADHD, with lower FA than the TD population being observed (Ikuta et al. 2014; Bathelt et al. 2019). The parahippocampal segment of the cingulum stems from the temporal lobe and fans out into the occipital lobe (Wu et al. 2016) and is involved in recognition memory (Metzler-Baddeley et al. 2012). The parahippocampal subdivision also includes projections from the amygdala, a structure that is often enlarged in TS (Kesler et al. 2004; Green et al. 2016) and which plays a role in recognizing facial expressions of fear, an area of deficit in TS (Skuse et al. 2005). The cingulum as a whole is one of the last fasciculi to develop fully, with changes seen in the tract until nearly the third decade of life (Tamnes et al. 2010a; Lebel et al. 2019). The parahippocampal segment may have an even more prolonged developmental trajectory as Tamnes et al. (2010a) reported that FA in the parahippocampal region reached 90% of its peak value around 35 years of age. Because the developmental period is so long, this tract may be especially susceptible to damage or altered growth as there are more opportunities for disruption in development. Sex differences in RD in this tract have been reported previously in a sample of older children and young adults, though the direction of effect was not specified (Tamnes et al. 2010b).

The motor portion of the corpus callosum, connecting the primary motor cortices in both hemispheres, also presents a masculinizing effect (X0 = XY > XX) at the global level, but in this case, AD is greater in TD males and TS females than in TD females, leading us to the opposite conclusion; this fasciculus is more mature in TD females than TS females or TD males. Examination of local statistics shows that the overall masculinizing pattern largely reflects difference near the center of the tract. There is also a small region near the left hemisphere periphery that follows a PAR pattern. Individuals with TS have documented difficulties with motor functions in terms of speed and number of motions required for a task (Nijhuis-van der Sanden et al. 2002, 2003). The greater the spatial processing on these tasks, the poorer performance observed in motor control (Ross et al. 1996). However, the deficits in motor function do not appear to be linked to the cognitive profile observed in TS (Der et al. 2000).

The tapetum is a segment of the corpus callosum that extends laterally on either side into the temporal lobe (Pizzini et al. 2008). The tapetum has been reported in the literature for TS as having aberrant diffusivity (Yamagata et al. 2012; Xie et al. 2015). This aligns with the cognitive profile of TS as the tapetum is an important contributor to visuospatial functioning (Tusa and Ungerleider 1985), and social and communicative functioning (Hanaie et al. 2014). In general, our results for FA are in keeping with the existing literature with TD females having greater overall fiber integrity than TS females at multiple locations along the tract. In the literature, TS is associated with lower FA in the tapetum (Yamagata et al. 2012; Villalon et al. 2013). However, there were two locations, one in the far left periphery of the tract and one in the far right periphery of the tract, where TS females had higher FA than the control groups. For AD, examination of local statistics revealed two regions where females with TS differed significantly from XX females. In one region, AD was higher in TS females, suggesting delayed development; in the other, AD was lower in TS females, suggesting faster development. Although not statistically significant, similar differences were observed when comparing TS females to XY males, suggesting PAR-mediated effects on both FA and RD in this tract.

Corticofugal tracts include corticoefferent and corticopetal fiber groups that interconnect the cerebral cortex, corona radiata, internal capsule, cerebral peduncles, pontine nuclei, and/or the brainstem. For AD and RD, the left motor corticofugal tract follows the same pattern we see in the right cingulum adjoining the hippocampus, with TD females showing slower maturation than TD males and females with TS. Significant group differences were also observed for FA. However, the primary pattern of differences for FA is more in keeping with a PAR effect than a masculinization effect with TS females having higher FA than both TD males and TD females. The corticofugal tracts travel through the internal capsule, which shows lower FA in TS girls than TD girls (Holzapfel et al. 2006; Yamagata et al. 2012), suggesting that the phenotype we observed does not persist into later childhood and may, in fact, reverse. Interestingly, sex differences in the internal capsule are present in middle age, with higher FA in males than females, suggesting that the trajectories of XX females and XY males eventually diverge (Takao et al. 2014). The relationship we observed in the left motor corticofugal tract could also be a contributor to the motor phenotype observed in TS.

Corticothalamic fiber tracts are white matter pathways that radiate from the thalamus to the cerebral cortex, via the internal capsule and corona radiata. They are also known as thalamic radiations. Beginning in the thalamus, the premotor corticothalamic tract travels to the premotor regions of the frontal cortex, which function in the planning and organization of movements. Like many of our other findings, the right premotor corticothalamic tract appears to be masculinized in TS and exhibits earlier maturation (XX > X0 = XY; AD). As previously discussed for the corticofugal tract, lower FA in the internal capsule was observed in young girls with TS when compared to TD girls (Holzapfel et al. 2006; Yamagata et al. 2012).

The left IFOF connects orbitofrontal cortex and the inferior frontal gyrus to the occipital lobe. We observed substantial regional heterogeneity in how group membership affected diffusivity in the IFOF. For AD, we observed a potential sex difference in the anterior IFOF with TS females having significantly lower AD than XY males, but similar AD to females, suggesting faster development of this area in XY males. In the posterior IFOF, we identified a region where AD was increased in TS compared to both XX females and XY males, indicating a potential PAR effect. For FA, we observed potential sex differences in the anterior frontal lobe and midsection where females (both XX and XO) had greater tract integrity than XY males. There was also a section in the posterior frontal lobe that followed a PAR pattern with TS showing more integrity than both TD males and females. Finally, we observed a possible masculinization effect on a section of the posterior IFOF where TS females had greater tract integrity compared to XX females but were similar to XY males. The IFOF is involved in attentional control and may contribute to attentional difficulties in ADHD (Shaw et al. 2015), so the fact that we observed delayed maturation of AD in this fasciculus in TS could partially explain the increased vulnerability of girls with TS to ADHD.

The left inferior longitudinal fasciculus connects the temporal and occipital lobes. Group differences in this tract for both FA and RD seemed to reflect hemizygosity in the PAR with TS individuals exhibiting greater myelination and higher tract integrity compared to both males and females. In ASD, reduced FA has been observed in the ILF (Jou et al. 2011; Koldewyn et al. 2014; Boets et al. 2018), which is the opposite of what we have found for infants with TS. However, in a study in girls with TS from ages 7 to 14, a reduction in FA was observed in the ILF (Villalon et al. 2013). As with the right frontotemporal segment of the arcuate fasciculus, initially accelerated development of this tract in TS may be followed by a slowing of development, allowing typically developing girls to overtake girls with TS.

Finally, the right optic tract transports visual information from the optic chiasm to the right lateral geniculate body as a part of the visual pathway. Global test statistics suggest that group differences in the right optic tract primarily reflect a sex difference in FA with both TS females and XX females differing significantly from XY males, but not differing from each other. Examination of local test statistics reveals additional complexity. FA differences in the anterior part of this tract are challenging to classify into one of the three patterns with significant differences between TS females and XY males (X0 > XY), but small and nonsignificant differences between TS and XX females and between XX females and XY males. The posterior part of this tract shows a pattern of masculinized FA (X0 = XY > XX). To our knowledge, this tract has not been examined in adolescence or adulthood in TS, nor are we aware of any studies reporting a sex difference in this tract.

The current study has many strengths including the use of advanced image acquisition and analysis techniques optimized for studies of the infant brain. The inclusion of both male and female control groups provides unique insights into possible underlying mechanisms. The developmental period we have chosen also alleviates the issue of the complications inherent in treatment of TS with hormone therapy used to induce puberty, which has been demonstrated to cause changes in connectivity (Peper et al. 2011). A possible limitation to the study is our moderate sample size, which may cause us to be underpowered to detect subtle differences in functional connectivity and white matter microstructure. Another limitation is the relatively short duration of the rs-fMRI scan (3 min), which may have contributed to the negative findings of functional connectivity differences in this report. However, previous work has shown that functional connectivity is reliable and stable for scans between 3 and 12 min for group-level characterizations and comparisons (Van Dijk et al. 2010; Braun et al. 2012) and we have used 3-min rs-fMRI data to robustly characterize and detect functional connectivity changes in infants of similar ages and young children (Salzwedel et al. 2019; Chen et al. 2021a, 2021b; Liu et al. 2021). Nevertheless, there remains a debate on the optimal length of rs-fMRI data acquisition, especially for individualized characterizations (Gordon et al. 2017), so future studies with longer rs-fMRI acquisitions are needed to validate findings in this study. It is difficult to ascertain whether the current sample is adequately powered to detect differences in diffusivity indices in the SLF reported in older patients for several reasons: (i) Prior studies did not routinely report effect sizes, and (ii) prior studies used different methods. Regarding methodological differences between studies, our analysis framework is based on streamline tractography performed in an unbiased average DTI tensor atlas developed specifically for 1-year-old children. Tracts are parameterized by length to reveal diffusion properties as a function of location along the tracts, and functional statistical analysis methods are used to compute local and global statistics. Holzapfel et al. (2006) used a voxel-wise approach within a white matter mask with cluster-based statistical testing for FA only. Villalon et al. (2013) also used a voxel-wise approach in which FA maps from each individual subject were thresholded at FA > 0.2 to exclude contributions from nonwhite matter, and FA, AD, RD, and MD were compared. They then overlaid the significant areas in the space of the subject’s tractography in order to identify tracts intersecting regions of significance. However, they did not run any direct statistical comparisons using the tractography data. Yamagata et al. (2012) used Tract-Based Spatial Statistics (TBSS) as their primary approach followed by a confirmatory ROI-based analysis. They also conducted a post hoc fiber-tracking analysis of the SLF. This analysis is most comparable to ours, but differs in several key ways. First, they compared the average diffusivity values for the entire track between groups, while we look along the tract, providing greater specificity. Second, they are doing tractography in each individual brain, while we perform our tracking on an atlas, which provides more robust results in infants and generally has better signal than individual tractography. We note that ROI and voxel-based analyses can be influenced by partial volume effects, while TBSS and tract-based analyses are not, and this could also contribute to differences across studies.

## Conclusion

In conclusion, this study provides novel information about functional connectivity and white matter microstructure in infants with TS. By comparing our results to the existing literature in older children, adolescents, and adults with TS, we have begun to construct a developmental model of this condition. Our results indicate that disrupted connectivity between frontal cortex and parieto-occipital cortex, as indexed by diffusivity measures in the left SLF and resting-state connectivity between the right precentral gyrus and right and left calcarine cortex, right and left lingual cortex, and right supramarginal cortex, is not present in late infancy. Early intervention could potentially prevent/minimize these brain phenotypes from emerging and lessen the cognitive impact of this genetic disorder in later stages of development. However, our exploratory studies revealed diffusivity differences in other white matter tracts during infancy. Some of these differences appear to persist into adulthood, while others appear to be specific to this developmental period. It is suggested that future studies look longitudinally at structural and functional connectivity in girls with TS, perhaps targeting the infant, toddler, and preschool developmental epoch, in order to better understand brain development in this group, identify biomarkers for later cognitive challenges, and identify critical periods for intervention.

## Supplementary Material

A2_supplement_submitted_bhae351

Supplementary_Table_2_ANCOVA_exploratory_rsfMRI_08012024_bhae351

Supplementary_Table_3_Pairwise_comps_exploratory_rsfMRI_08012024_bhae351

## Data Availability

Data is available on request by contacting the corresponding author and may require IRB approval and a data sharing agreement.
