# Supplementary material for: White matter microstructure and functional connectivity in the brains of infants with Turner syndrome
Source: Cereb Cortex. 2024 Sep 10;34(9):bhae351. doi: 10.1093/cercor/bhae351 (PMC11387115; doi:10.1093/cercor/bhae351)
Supplement: A2_supplement_submitted_bhae351 [file a2_supplement_submitted_bhae351.docx]

| **Region** | **LS (SE) TS** | **LS (SE) Female** | **LS (SE) Male** | **Uncorrected p-value** | **FDR Corrected p-value** |
| --- | --- | --- | --- | --- | --- |
| Right calcarine cortex | -0.053 (0.092) | 0.197 (0.075) | 0.168 (0.078) | .0128 | 0.128 |
| Left calcarine cortex | -0.054 (0.087) | 0.189 (0.071) | 0.122 (0.075) | 0.139 | 0.139 |
| Right lingual cortex | 0.016 (0.082) | 0.297 (0.067) | 0.231 (0.071) | 0.051 | 0.514 |
| Left lingual cortex | 0.056 (0.081) | 0.217 (0.066) | 0.163 (0.069) | 0.358 | 0.358 |
| Right supramarginal gyrus | 0.372 (0.097) | 0.323 (0.079) | 0.246 (0.083) | 0.589 | 0.589 |

**Supplementary Table 1. One-way ANCOVA results for resting-state functional connectivity between the right precentral gyrus and five occipital-parietal regions showing reduced volumes in infants with TS without using global signal regression.**

**Supplementary Table 2. One-way ANCOVA results for exploratory resting-state functional connectivity analyses.** See separate excel file.

**Supplementary Table 3. Pairwise comparisons for exploratory resting-state functional connectivity analyses.** See separate excel file.

**Supplementary Table 4 One-way ANCOVA results for resting-state functional connectivity between the right supramarginal gyrus and the two regions showing statistical significance after FDR correction in our 6 by 90 calculations.**

| **Region** | **LS (SE) TS** | **LS (SE) Female** | **LS (SE) Male** |
| --- | --- | --- | --- |
| Left Insula | 0.426 (0.084) | -0.083 (0.068) | -0.0817 (0.072) |
| Right Putamen | 0.249 (0.079) | -0.09 (0.064) | -0.257 (0.068) |

**Supplementary Table 5. The 46 tracts that passed quality control with global omnibus and FDR p-values and p-values for axial diffusivity (AD), radial diffusivity (RD), and fractional anisotropy (FA).**

|  |  | Global_Omnibus | Global_FDR |  | AD | RD | FA |
| --- | --- | --- | --- | --- | --- | --- | --- |
| Arcuate, left, frontoparietal |  |  |  |  |  |  |  |
|  | Female control, Male control | 0.086 | 0.16483 |  | 0.163 | 0.23 | 0.064 |
|  | Female control, Turner syndrome | 0.489 | 0.5442097 |  | 0.751 | 0.475 | 0.129 |
|  | Male control, Turner syndrome | 0.138 | 0.226714 |  | 0.238 | 0.229 | 0.192 |
| Arcuate, left, frontotemporal |  |  |  |  |  |  |  |
|  | Female control, Male control | 0.004 | 0.02208 |  | 0 | 0.557 | 0.505 |
|  | Female control, Turner syndrome | 0.536 | 0.5870476 |  | 0.39 | 0.499 | 0.939 |
|  | Male control, Turner syndrome | 0.328 | 0.4066726 |  | 0.398 | 0.363 | 0.169 |
| Arcuate, left, temporoparietal |  |  |  |  |  |  |  |
|  | Female control, Male control | 0.0001 | 0.00092 |  | 0.098 | 0 | 0.051 |
|  | Female control, Turner syndrome | 0.02 | 0.069 |  | 0.25 | 0.113 | 0.056 |
|  | Male control, Turner syndrome | 0.046 | 0.1075932 |  | 0.015 | 0.15 | 0.3 |
| Arcuate, right, fronotparietal |  |  |  |  |  |  |  |
|  | Female control, Male control | 0.076 | 0.1518 |  | 0.137 | 0.029 | 0.358 |
|  |  |  |  |  |  |  |  |
|  | Female control, Turner syndrome | 0.057 | 0.1191818 |  | 0.103 | 0.418 | 0.145 |
|  | Male control, Turner syndrome | 0.123 | 0.2139 |  | 0.03 | 0.063 | 0.056 |
| Arcuate right, frontotemporal |  |  |  |  |  |  |  |
|  | Female control, Male control | 0.026 | 0.0763404 |  | 0.062 | 0.013 | 0.255 |
|  | Female control, Turner syndrome | 0.007 | 0.0333103 |  | 0.009 | 0.224 | 0.078 |
|  | Male control, Turner syndrome | 0.387 | 0.4557479 |  | 0.329 | 0.725 | 0.319 |
| Cingulate gyrus, left |  |  |  |  |  |  |  |
|  | Female control, Male control | 0.137 | 0.2267143 |  | 0.103 | 0.193 | 0.51 |
|  | Female control, Turner syndrome | 0.39 | 0.4557479 |  | 0.287 | 0.513 | 0.715 |
|  | Male control, Turner syndrome | 0.387 | 0.4557479 |  | 0.78 | 0.441 | 0.569 |
| Cingulate gyrus, right |  |  |  |  |  |  |  |
|  | Female control, Male control | 0.023 | 0.0721364 |  | 0.031 | 0.046 | 0.332 |
|  | Female control, Turner syndrome | 0.047 | 0.1081 |  | 0.052 | 0.25 | 0.539 |
|  | Male control, Turner syndrome | 0.708 | 0.7291343 |  | 0.501 | 0.879 | 0.977 |
| Cingulum adjoining hippocampus, left |  |  |  |  |  |  |  |
|  | Female control, Male control | 0.002 | 0.0131429 |  | 0.154 | 0.007 | 0.078 |
|  | Female control, Turner syndrome | 0.018 | 0.0671351 |  | 0.24 | 0.009 | 0.035 |
|  | Male control, Turner syndrome | 0.22 | 0.31625 |  | 0.43 | 0.399 | 0.058 |
| Cingulum adjoining hippocampus, right |  |  |  |  |  |  |  |
|  | Female control, Male control | 0.0001 | 0.00092 |  | 0.004 | 0 | 0.526 |
|  | Female control, Turner syndrome | 0.0001 | 0.00092 |  | 0.001 | 0.002 | 0.197 |
|  | Male control, Turner syndrome | 0.466 | 0.5228293 |  | 0.608 | 0.87 | 0.899 |
| Corpus callosum, body |  |  |  |  |  |  |  |
|  | Female control, Male control | 0.001 | 0.0072632 |  | 0.004 | 0.144 | 0.495 |
|  | Female control, Turner syndrome | 0.159 | 0.2493409 |  | 0.217 | 0.631 | 0.466 |
|  | Male control, Turner syndrome | 0.386 | 0.4557479 |  | 0.168 | 0.91 | 0.723 |
| Corpus callosum, genu |  |  |  |  |  |  |  |
|  | Female control, Male control | 0.005 | 0.0265385 |  | 0.004 | 0.106 | 0.074 |
|  | Female control, Turner syndrome | 0.188 | 0.2882667 |  | 0.627 | 0.378 | 0.249 |
|  | Male control, Turner syndrome | 0.333 | 0.4066726 |  | 0.318 | 0.639 | 0.893 |
| Corpus callosum, motor |  |  |  |  |  |  |  |
|  | Female control, Male control | 0.013 | 0.0543636 |  | 0.099 | 0.863 | 0.641 |
|  | Female control, Turner syndrome | 0.007 | 0.0333103 |  | 0.027 | 0.426 | 0.261 |
|  | Male control, Turner syndrome | 0.607 | 0.6443538 |  | 0.419 | 0.584 | 0.477 |
| Corpus callosum, parietal |  |  |  |  |  |  |  |
|  | Female control, Male control | 0.0001 | 0.00092 |  | 0 | 0.007 | 0.112 |
|  | Female control, Turner syndrome | 0.052 | 0.1104 |  | 0.173 | 0.088 | 0.06 |
|  | Male control, Turner syndrome | 0.656 | 0.6858182 |  | 0.59 | 0.381 | 0.145 |
| Corpus callosum, premotor |  |  |  |  |  |  |  |
|  | Female control, Male control | 0.148 | 0.2402824 |  | 0.334 | 0.384 | 0.176 |
|  | Female control, Turner syndrome | 0.199 | 0.2952903 |  | 0.067 | 0.861 | 0.361 |
|  | Male control, Turner syndrome | 0.157 | 0.2490345 |  | 0.074 | 0.83 | 0.614 |
| Corpus callosum, rostrum |  |  |  |  |  |  |  |
|  | Female control, Male control | 0.023 | 0.0721364 |  | 0.148 | 0.386 | 0.023 |
|  | Female control, Turner syndrome | 0.108 | 0.1910769 |  | 0.08 | 0.33 | 0.121 |
|  | Male control, Turner syndrome | 0.445 | 0.51175 |  | 0.037 | 0.685 | 0.339 |
| Corpus callosum, splenium |  |  |  |  |  |  |  |
|  | Female control, Male control | 0.009 | 0.0388125 |  | 0.033 | 0.042 | 0.087 |
|  | Female control, Turner syndrome | 0.021 | 0.0706829 |  | 0.016 | 0.013 | 0.022 |
| Corpus callosum, tapetum |  |  |  |  |  |  |  |
|  | Female control, Male control | 0.312 | 0.4038716 |  | 0.84 | 0.941 | 0.104 |
|  | Female control, Turner syndrome | 0.004 | 0.02208 |  | 0.042 | 0.241 | 0.011 |
|  | Male control, Turner syndrome | 0.051 | 0.1104 |  | 0.178 | 0.505 | 0.09 |
| Corticofugal, left, motor |  |  |  |  |  |  |  |
|  | Female control, Male control | 0.0001 | 0.00092 |  | 0.0001 | 0.0001 | 0.654 |
|  | Female control, Turner syndrome | 0.0001 | 0.00092 |  | 0.0001 | 0.0001 | 0.021 |
|  | Male control, Turner syndrome | 0.0001 | 0.00092 |  | 0.004 | 0.087 | 0.0001 |
| Corticofugal, left, parietal |  |  |  |  |  |  |  |
|  | Female control, Male control | 0.007 | 0.0333103 |  | 0.055 | 0.012 | 0.233 |
|  | Female control, Turner syndrome | 0.099 | 0.1774286 |  | 0.128 | 0.383 | 0.105 |
|  | Male control, Turner syndrome | 0.002 | 0.0131429 |  | 0.097 | 0.029 | 0.02 |
| Corticofugal, right, motor |  |  |  |  |  |  |  |
|  | Female control, Male control | 0.033 | 0.0843333 |  | 0.015 | 0.231 | 0.517 |
|  | Female control, Turner syndrome | 0.045 | 0.107069 |  | 0.089 | 0.811 | 0.176 |
| Corticofugal, right, parietal |  |  |  |  |  |  |  |
|  | Female control, Male control | 0.023 | 0.0721364 |  | 0.048 | 0.058 | 0.472 |
|  | Female control, Turner syndrome | 0.263 | 0.3537087 |  | 0.205 | 0.42 | 0.833 |
|  | Male control, Turner syndrome | 0.02 | 0.069 |  | 0.103 | 0.861 | 0.034 |
| Corticoreticular, left |  |  |  |  |  |  |  |
|  | Female control, Male control | 0.0001 | 0.00092 |  | 0.001 | 0 | 0.351 |
|  | Female control, Turner syndrome | 0.062 | 0.1277015 |  | 0.374 | 0.086 | 0.016 |
|  | Male control, Turner syndrome | 0.463 | 0.5228293 |  | 0.188 | 0.946 | 0.285 |
| Corticoreticular, right |  |  |  |  |  |  |  |
|  | Female control, Male control | 0.052 | 0.1104 |  | 0.059 | 0.057 | 0.352 |
|  | Female control, Turner syndrome | 0.051 | 0.1104 |  | 0.072 | 0.164 | 0.19 |
|  | Male control, Turner syndrome | 0.314 | 0.4038716 |  | 0.432 | 0.509 | 0.132 |
| Corticospinal, right |  |  |  |  |  |  |  |
|  | Female control, Male control | 0.003 | 0.018 |  | 0.03 | 0.054 | 0.094 |
|  | Female control, Turner syndrome | 0.352 | 0.4261053 |  | 0.163 | 0.849 | 0.567 |
|  | Male control, Turner syndrome | 0.019 | 0.069 |  | 0.006 | 0.212 | 0.387 |
|  |  |  |  |  |  |  |  |
| Corticothalamic, left, motor |  |  |  |  |  |  |  |
|  | Female control, Male control | 0.319 | 0.4038716 |  | 0.29 | 0.241 | 0.204 |
|  | Female control, Turner syndrome | 0.319 | 0.4038716 |  | 0.29 | 0.241 | 0.204 |
|  | Male control, Turner syndrome | 0.853 | 0.8592263 |  | 0.892 | 0.962 | 0.882 |
| Corticothalamic, left, parietal |  |  |  |  |  |  |  |
|  | Female control, Male control | 0.0001 | 0.00092 |  | 0.009 | 0.005 | 0.192 |
|  | Female control, Turner syndrome | 0.688 | 0.7138647 |  | 0.506 | 0.745 | 0.367 |
|  | Male control, Turner syndrome | 0.218 | 0.31625 |  | 0.308 | 0.774 | 0.141 |
| Corticothalamic, left, prefrontal |  |  |  |  |  |  |  |
|  | Female control, Male control | 0.042 | 0.1016842 |  | 0.097 | 0.133 | 0.16 |
|  | Female control, Turner syndrome | 0.083 | 0.1613239 |  | 0.067 | 0.137 | 0.1 |
|  | Male control, Turner syndrome | 0.241 | 0.3359394 |  | 0.056 | 0.211 | 0.506 |
| Corticothalamic, left, premotor |  |  |  |  |  |  |  |
|  | Female control, Male control | 0.0001 | 0.00092 |  | 0 | 0.07 | 0.227 |
|  | Female control, Turner syndrome | 0.036 | 0.0887143 |  | 0.081 | 0.047 | 0.037 |
|  | Male control, Turner syndrome | 0.638 | 0.6720916 |  | 0.325 | 0.971 | 0.761 |
| Corticothalamic, left, superior |  |  |  |  |  |  |  |
|  | Female control, Male control | 0.0001 | 0.00092 |  | 0 | 0.019 | 0.206 |
|  | Female control, Turner syndrome | 0.193 | 0.2926813 |  | 0.599 | 0.196 | 0.133 |
|  | Male control, Turner syndrome | 0.845 | 0.8574265 |  | 0.539 | 0.8 | 0.901 |
| Corticothalamic, right, motor |  |  |  |  |  |  |  |
|  | Female control, Male control | 0.027 | 0.077625 |  | 0.01 | 0.619 | 0.039 |
|  | Female control, Turner syndrome | 0.031 | 0.0822692 |  | 0.008 | 0.405 | 0.167 |
|  | Male control, Turner syndrome | 0.871 | 0.871 |  | 0.892 | 0.865 | 0.911 |
| Corticothalamic, right, parietal |  |  |  |  |  |  |  |
|  | Female control, Male control | 0.0001 | 0.00092 |  | 0.003 | 0.001 | 0.176 |
|  | Female control, Turner syndrome | 0.393 | 0.4557479 |  | 0.343 | 0.224 | 0.592 |
|  | Male control, Turner syndrome | 0.136 | 0.2267143 |  | 0.102 | 0.275 | 0.054 |
| Corticothalamic, right, prefrontal |  |  |  |  |  |  |  |
|  | Female control, Male control | 0.241 | 0.3359394 |  | 0.263 | 0.497 | 0.594 |
|  | Female control, Turner syndrome | 0.281 | 0.3728654 |  | 0.131 | 0.618 | 0.7 |
|  | Male control, Turner syndrome | 0.29 | 0.3811429 |  | 0.454 | 0.255 | 0.963 |
| Corticothalamic, right, premotor |  |  |  |  |  |  |  |
|  | Female control, Male control | 0.0001 | 0.00092 |  | 0 | 0.338 | 0.008 |
|  | Female control, Turner syndrome | 0.003 | 0.018 |  | 0.005 | 0.311 | 0.129 |
|  | Male control, Turner syndrome | 0.452 | 0.5155041 |  | 0.319 | 0.574 | 0.626 |
| Corticothalamic, right, superior |  |  |  |  |  |  |  |
|  | Female control, Male control | 0.0001 | 0.00092 |  | 0.001 | 0.507 | 0.042 |
|  | Female control, Turner syndrome | 0.025 | 0.075 |  | 0.019 | 0.435 | 0.251 |
|  | Male control, Turner syndrome | 0.507 | 0.559728 |  | 0.318 | 0.573 | 0.957 |
| Fornix, left |  |  |  |  |  |  |  |
|  | Female control, Male control | 0.015 | 0.0591429 |  | 0.101 | 0.037 | 0.011 |
|  | Female control, Turner syndrome | 0.014 | 0.0568235 |  | 0.055 | 0.054 | 0.064 |
|  | Male control, Turner syndrome | 0.229 | 0.3257938 |  | 0.161 | 0.13 | 0.363 |
| Fornix, right |  |  |  |  |  |  |  |
|  | Female control, Male control | 0.172 | 0.2666966 |  | 0.435 | 0.17 | 0.252 |
|  | Female control, Turner syndrome | 0.57 | 0.6145313 |  | 0.403 | 0.767 | 0.941 |
|  | Male control, Turner syndrome | 0.554 | 0.6019843 |  | 0.588 | 0.637 | 0.726 |
| Inferior fronto-occiptal fasciculus, left |  |  |  |  |  |  |  |
|  | Male control, Turner syndrome | 0.001 | 0.0072632 |  | 0.003 | 0.066 | 0.012 |
| Inferior fronto-occipital fasciculus, right |  |  |  |  |  |  |  |
|  | Female control, Male control | 0.089 | 0.1682466 |  | 0.127 | 0.11 | 0.126 |
|  | Female control, Turner syndrome | 0.153 | 0.2455116 |  | 0.184 | 0.772 | 0.275 |
|  | Male control, Turner syndrome | 0.264 | 0.3537087 |  | 0.181 | 0.229 | 0.251 |
| Inferior longitudinal fasciculus, left |  |  |  |  |  |  |  |
|  | Female control, Male control | 0.017 | 0.0651667 |  | 0.045 | 0.801 | 0.313 |
|  | Female control, Turner syndrome | 0.001 | 0.0072632 |  | 0.201 | 0.044 | 0.039 |
|  | Male control, Turner syndrome | 0.028 | 0.0788571 |  | 0.339 | 0.247 | 0.073 |
| Inferior longitudinal fasciculus, right |  |  |  |  |  |  |  |
|  | Female control, Male control | 0.031 | 0.0822692 |  | 0.106 | 0.031 | 0.139 |
|  | Female control, Turner syndrome | 0.05 | 0.1104 |  | 0.279 | 0.132 | 0.072 |
|  | Male control, Turner syndrome | 0.124 | 0.2139 |  | 0.589 | 0.118 | 0.273 |
| Optic tract, right |  |  |  |  |  |  |  |
|  |  |  |  |  |  |  |  |
|  | Female control, Turner syndrome | 0.035 | 0.0878182 |  | 0.406 | 0.211 | 0.388 |
|  | Male control, Turner syndrome | 0.008 | 0.0356129 |  | 0.145 | 0.06 | 0.007 |
| Optic radiation, left |  |  |  |  |  |  |  |
|  | Female control, Male control | 0.001 | 0.0072632 |  | 0.1 | 0 | 0.001 |
|  | Female control, Turner syndrome | 0.095 | 0.1748 |  | 0.935 | 0.033 | 0.031 |
|  | Male control, Turner syndrome | 0.246 | 0.33948 |  | 0.502 | 0.169 | 0.352 |
| Optic radiation, right |  |  |  |  |  |  |  |
|  | Female control, Male control | 0.0001 | 0.00092 |  | 0.001 | 0.005 | 0.048 |
|  | Female control, Turner syndrome | 0.072 | 0.1461176 |  | 0.065 | 0.013 | 0.188 |
|  | Male control, Turner syndrome | 0.806 | 0.8239111 |  | 0.55 | 0.928 | 0.528 |
| Superior longitudinal fasciculus, left |  |  |  |  |  |  |  |
|  | Female control, Male control | 0.099 | 0.1774286 |  | 0.068 | 0.31 | 0.654 |
|  | Female control, Turner syndrome | 0.585 | 0.625814 |  | 0.427 | 0.515 | 0.296 |
|  | Male control, Turner syndrome | 0.092 | 0.1715676 |  | 0.212 | 0.077 | 0.205 |
| Uncinate, left |  |  |  |  |  |  |  |
|  | Female control, Male control | 0.197 | 0.2952903 |  | 0.14 | 0.45 | 0.136 |
|  |  |  |  |  |  |  |  |
|  | Male control, Turner syndrome | 0.077 | 0.1518 |  | 0.057 | 0.067 | 0.182 |
| Uncinate, right |  |  |  |  |  |  |  |
|  | Female control, Male control | 0.13 | 0.2214815 |  | 0.027 | 0.669 | 0.039 |
|  | Female control, Turner syndrome | 0.21 | 0.3082979 |  | 0.094 | 0.471 | 0.202 |
|  | Male control, Turner syndrome | 0.332 | 0.4066726 |  | 0.231 | 0.443 | 0.467 |

**
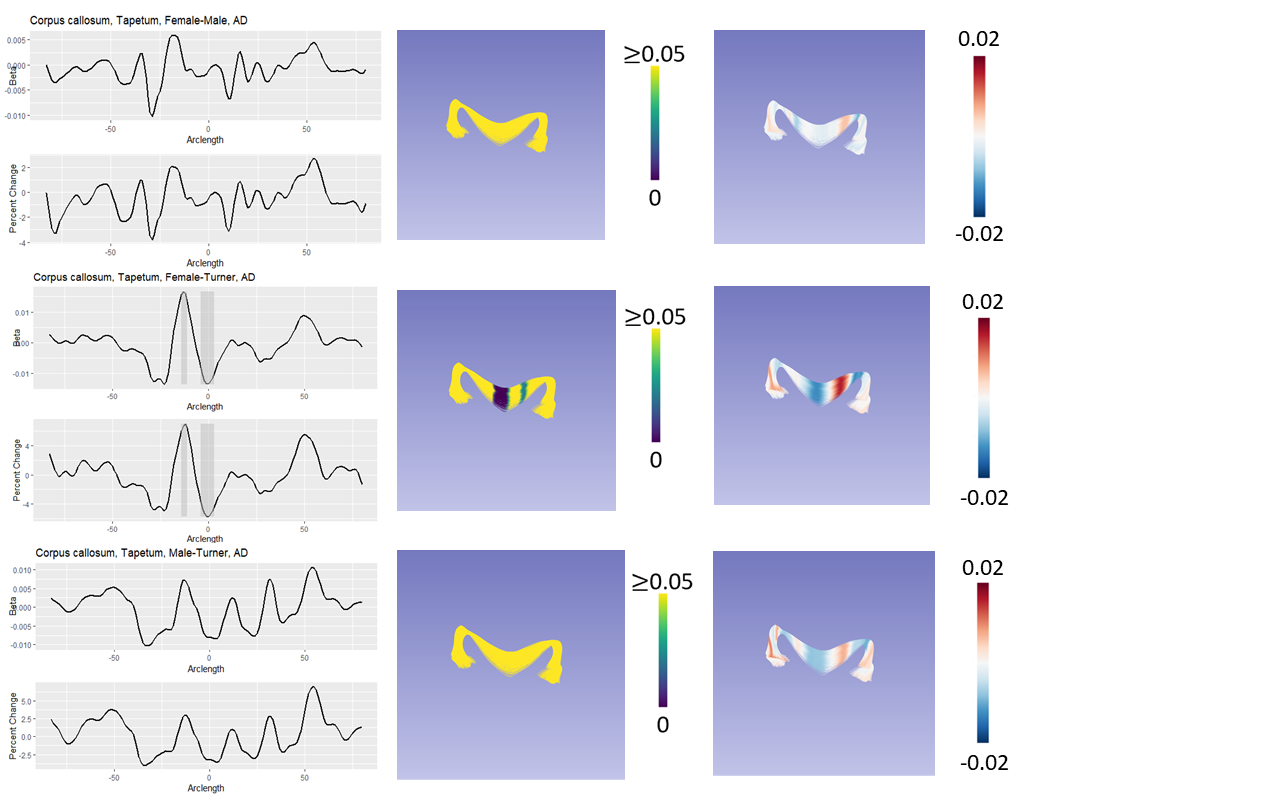
**

**Supplementary Figure 1 Model of DTI results for the tapetum portion of the corpus callosum for measures of axial diffusivity.** In each panel, the leftmost graph shows the beta value over the arclength of the fasciculus, with areas of local statistical significance highlighted in grey. Below that is the percent change in the beta value over time for that tract in the specified comparison for the specific diffusivity measure. The middlemost image in each panel shows an overlay of the p-values on the fasciculus, with regions of statistical significance showing a color other than yellow. Finally, the rightmost panel shows the beta values from the specific comparison with the model of the tract.

**
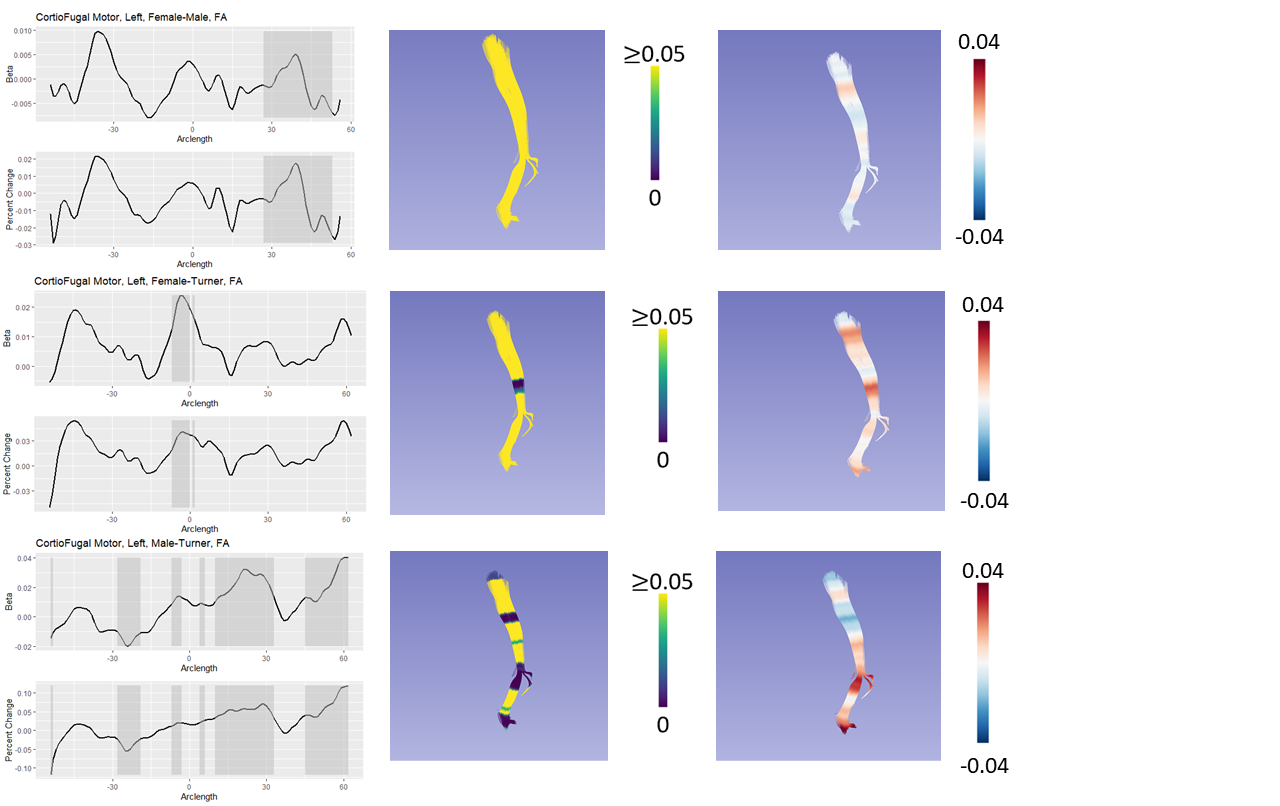
**

**Supplementary Figure 2 Model of DTI results for the left corticofugal tract for measures of fractional anisotropy.** In each panel, the leftmost graph shows the beta value over the arclength of the fasciculus, with areas of local statistical significance highlighted in grey. Below that is the percent change in the beta value over time for that tract in the specified comparison for the specific diffusivity measure. The middlemost image in each panel shows an overlay of the p-values on the fasciculus, with regions of statistical significance showing a color other than yellow. Finally, the rightmost panel shows the beta values from the specific comparison with the model of the tract.

**
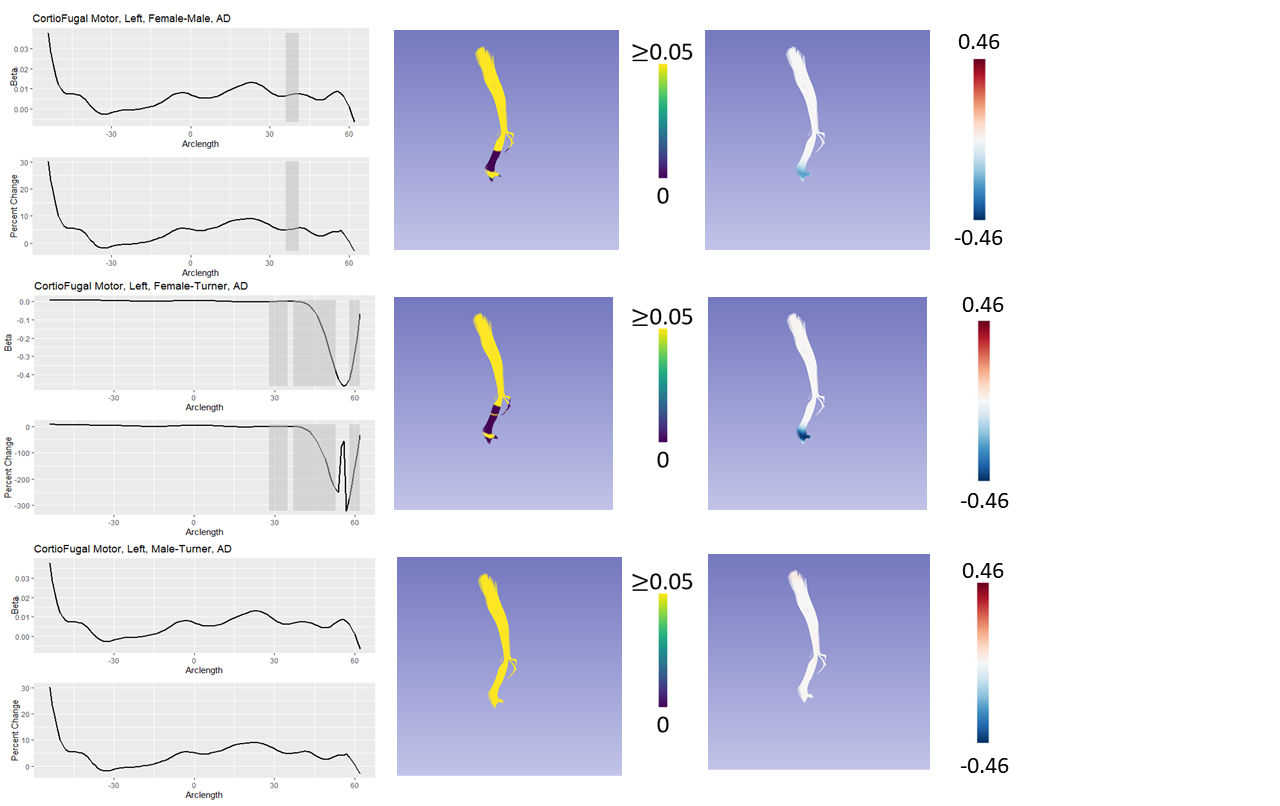
**

**Supplementary Figure 3** **Model of DTI results for the left motor corticofugal tract for measures of axial diffusivity.** In each panel, the leftmost graph shows the beta value over the arclength of the fasciculus, with areas of local statistical significance highlighted in grey. Below that is the percent change in the beta value over time for that tract in the specified comparison for the specific diffusivity measure. The middlemost image in each panel shows an overlay of the p-values on the fasciculus, with regions of statistical significance showing a color other than yellow. Finally, the rightmost panel shows the beta values from the specific comparison with the model of the tract.

**
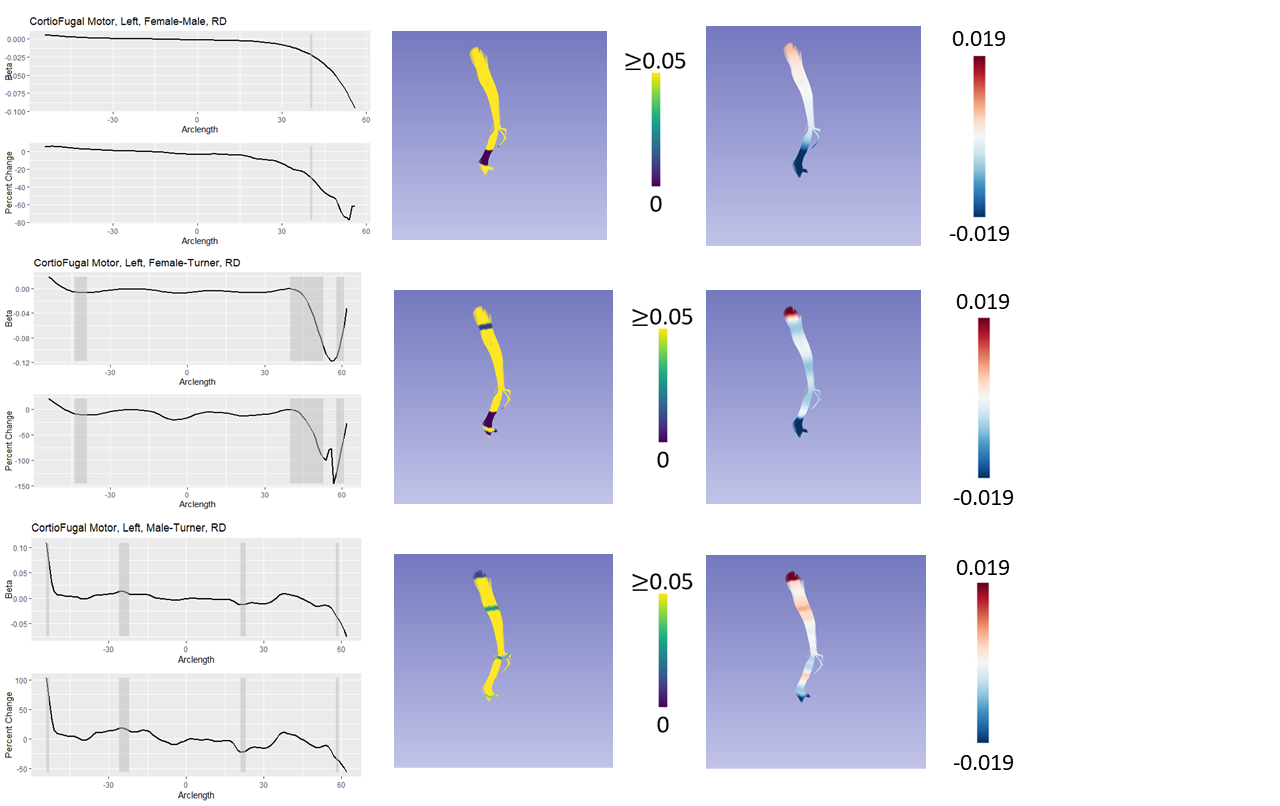
**

**Supplementary Figure 4** **Model of DTI results for the left motor corticofugal tract for measures of radial diffusivity.** In each panel, the leftmost graph shows the beta value over the arclength of the fasciculus, with areas of local statistical significance highlighted in grey. Below that is the percent change in the beta value over time for that tract in the specified comparison for the specific diffusivity measure. The middlemost image in each panel shows an overlay of the p-values on the fasciculus, with regions of statistical significance showing a color other than yellow. Finally, the rightmost panel shows the beta values from the specific comparison with the model of the tract.

**
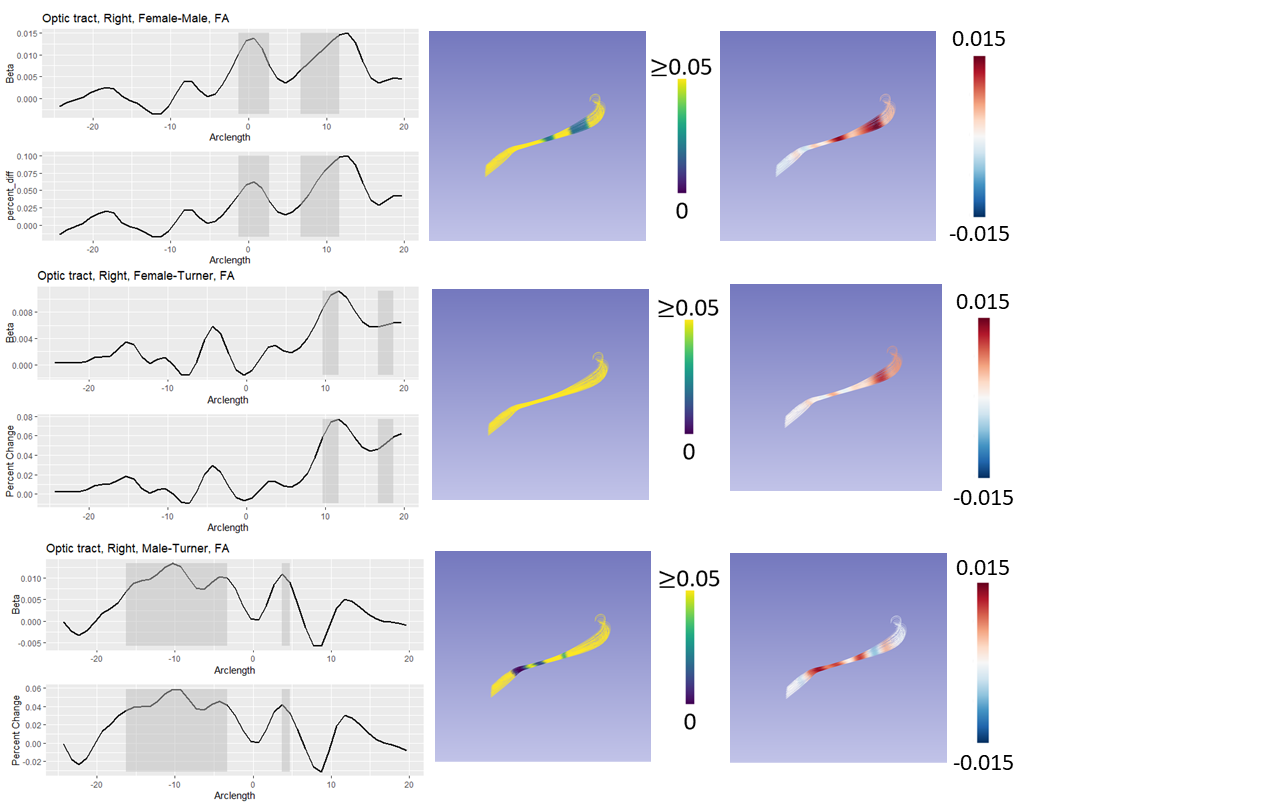
**

**Supplementary Figure 5** **Model of DTI results for the right optic tract for measures of fractional anisotropy.** In each panel, the leftmost graph shows the beta value over the arclength of the fasciculus, with areas of local statistical significance highlighted in grey. Below that is the percent change in the beta value over time for that tract in the specified comparison for the specific diffusivity measure. The middlemost image in each panel shows an overlay of the p-values on the fasciculus, with regions of statistical significance showing a color other than yellow. Finally, the rightmost panel shows the beta values from the specific comparison with the model of the tract.

**
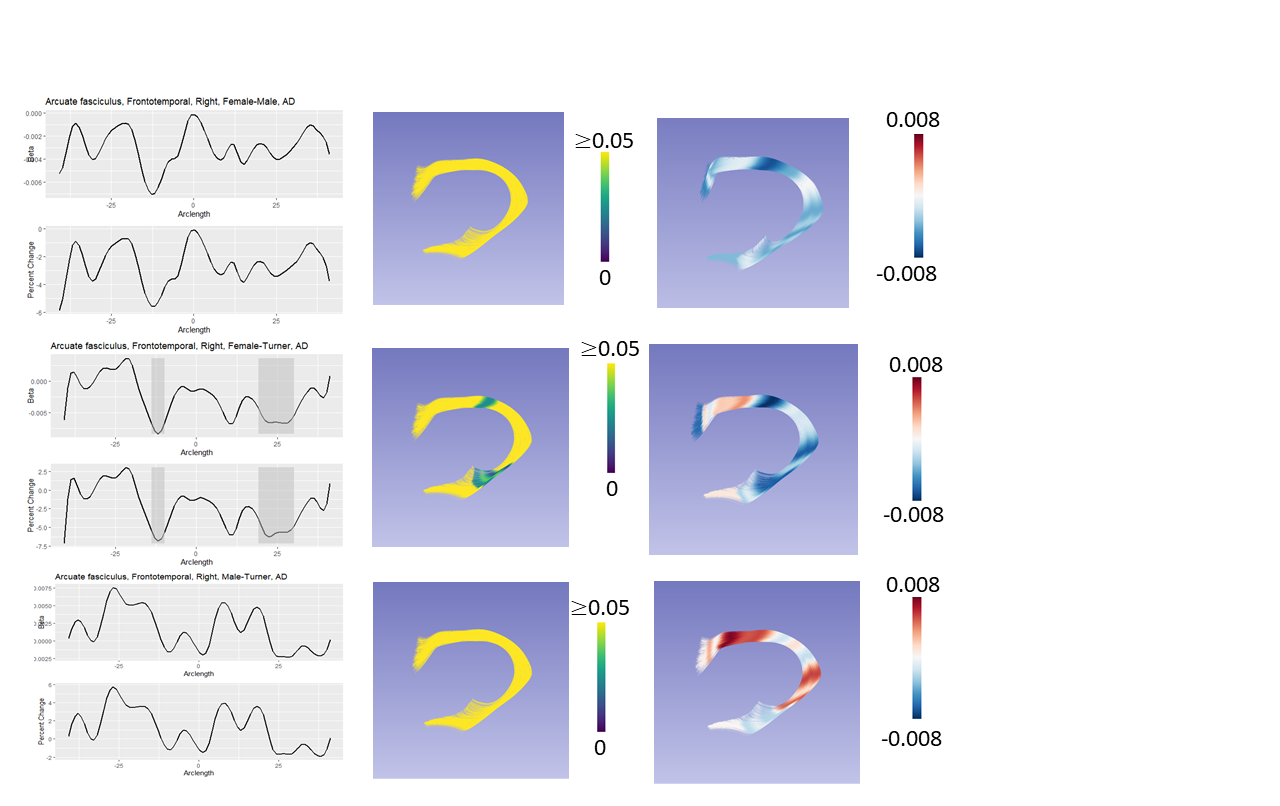
**

**Supplementary Figure 6** **Model of DTI results for right frontotemporal region of the arcuate fasciculus for measures of axial diffusivity.** In each panel, the leftmost graph shows the beta value over the arclength of the fasciculus, with areas of local statistical significance highlighted in grey. Below that is the percent change in the beta value over time for that tract in the specified comparison for the specific diffusivity measure. The middlemost image in each panel shows an overlay of the p-values on the fasciculus, with regions of statistical significance showing a color other than yellow. Finally, the rightmost panel shows the beta values from the specific comparison with the model of the tract.

**
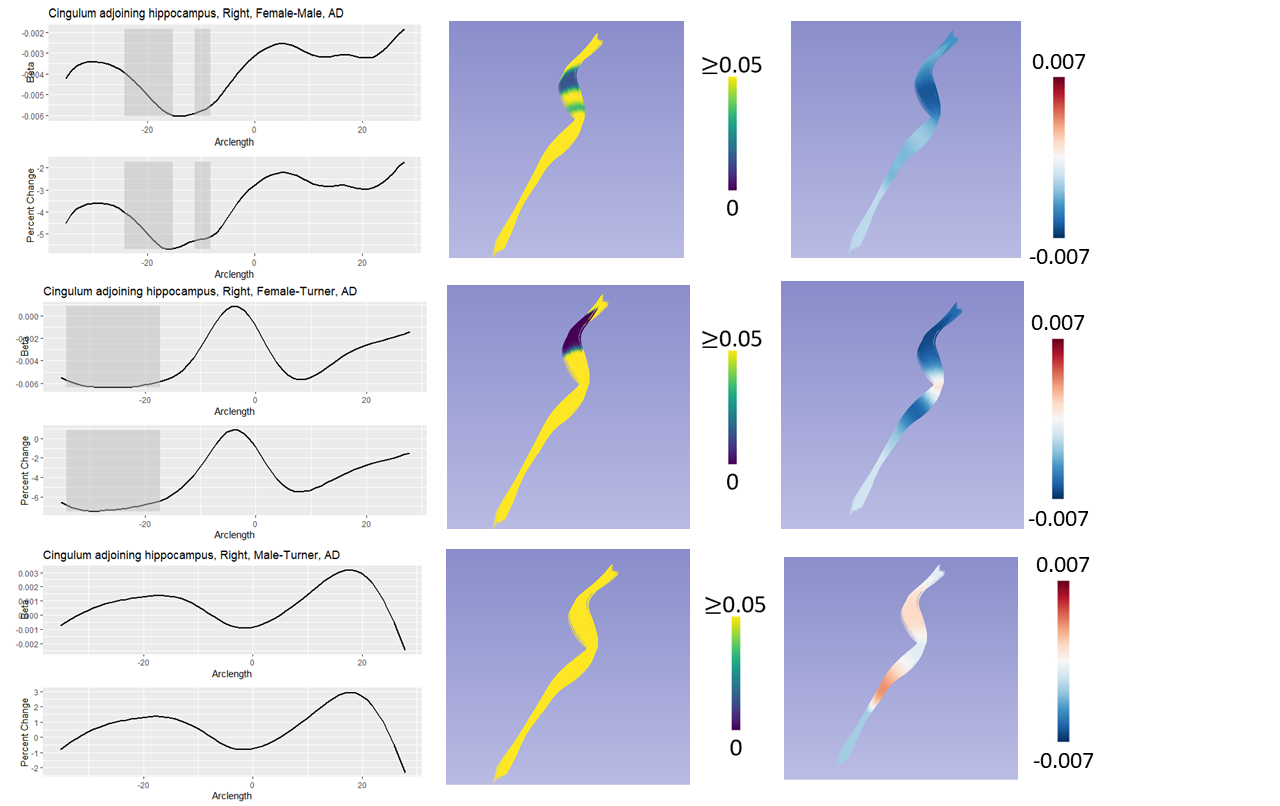
**

**Supplementary Figure 7** **Model of DTI results for the cingulum adjoining the hippocampus for measures of axial diffusivity.** In each panel, the leftmost graph shows the beta value over the arclength of the fasciculus, with areas of local statistical significance highlighted in grey. Below that is the percent change in the beta value over time for that tract in the specified comparison for the specific diffusivity measure. The middlemost image in each panel shows an overlay of the p-values on the fasciculus, with regions of statistical significance showing a color other than yellow. Finally, the rightmost panel shows the beta values from the specific comparison with the model of the tract.

**
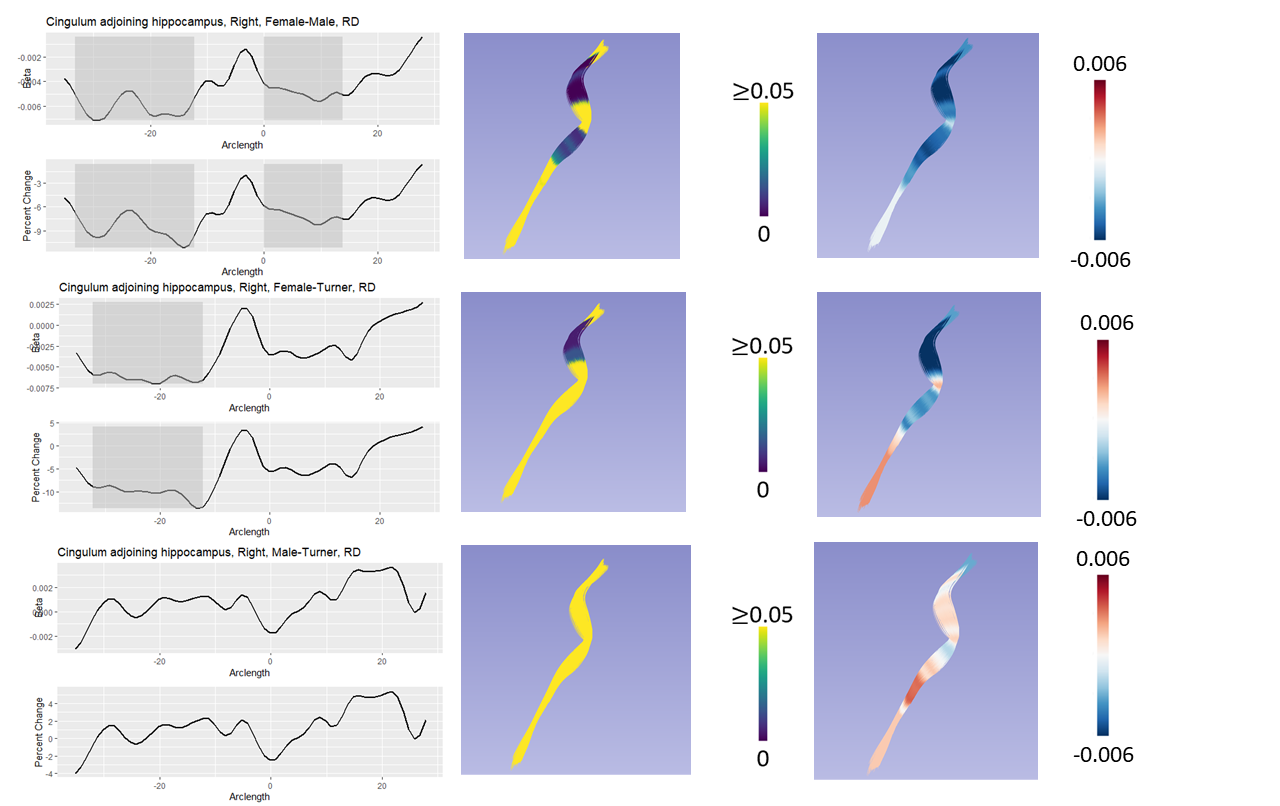
**

**Supplementary Figure 8** **Model of DTI results for the cingulum adjoining the hippocampus for measures of radial diffusivity.** In each panel, the leftmost graph shows the beta value over the arclength of the fasciculus, with areas of local statistical significance highlighted in grey. Below that is the percent change in the beta value over time for that tract in the specified comparison for the specific diffusivity measure. The middlemost image in each panel shows an overlay of the p-values on the fasciculus, with regions of statistical significance showing a color other than yellow. Finally, the rightmost panel shows the beta values from the specific comparison with the model of the tract.

**
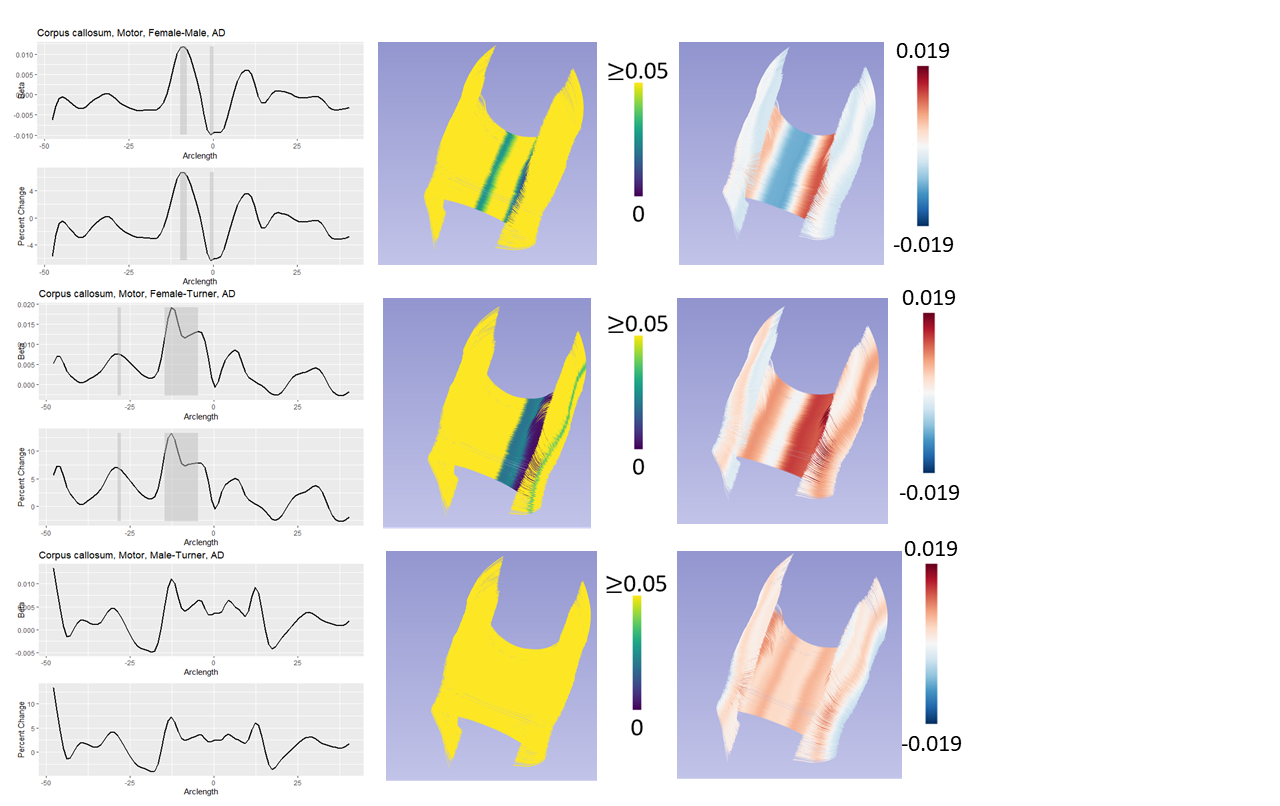
**

**Supplementary Figure 9 Model of DTI results for the motor bundle of the corpus callosum for measures of axial diffusivity.** In each panel, the leftmost graph shows the beta value over the arclength of the fasciculus, with areas of local statistical significance highlighted in grey. Below that is the percent change in the beta value over time for that tract in the specified comparison for the specific diffusivity measure. The middlemost image in each panel shows an overlay of the p-values on the fasciculus, with regions of statistical significance showing a color other than yellow. Finally, the rightmost panel shows the beta values from the specific comparison with the model of the tract.

**
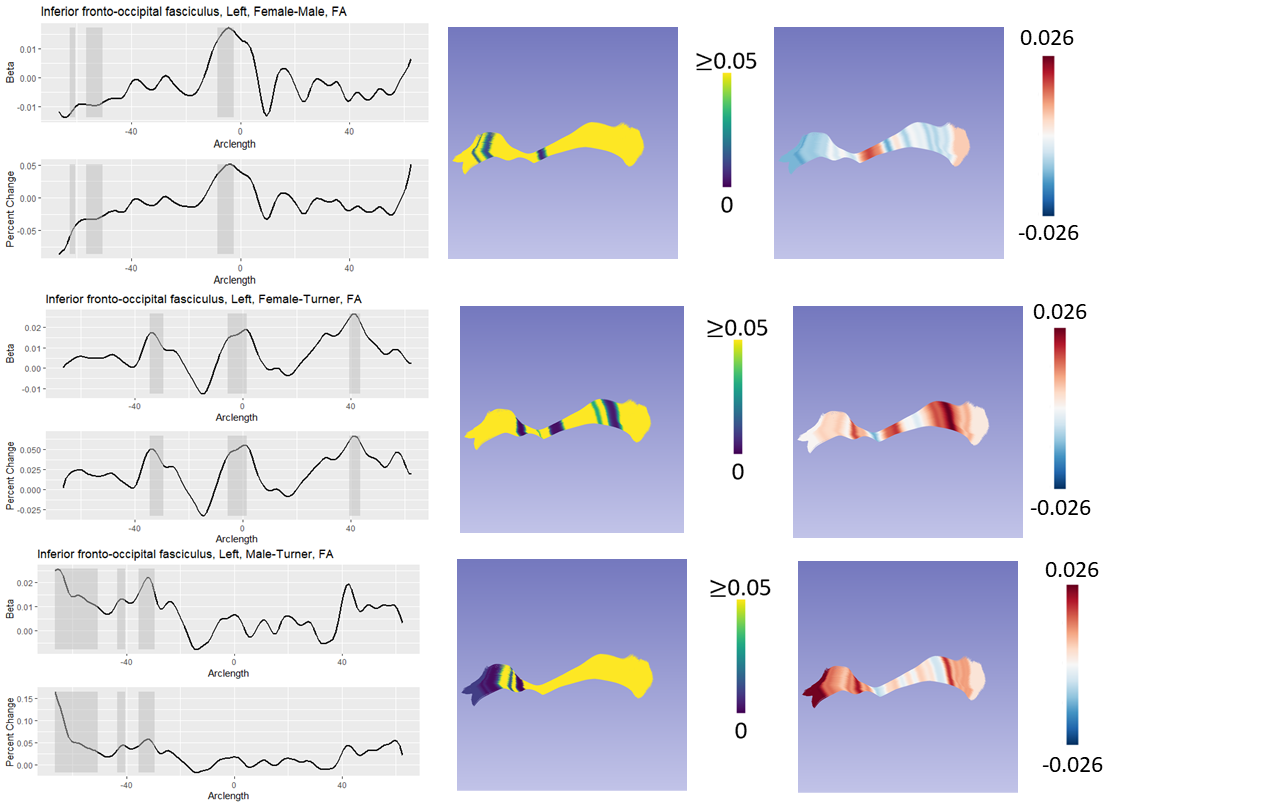
**

**Supplementary Figure 10** **Model of DTI results for the left inferior fronto-occipital fasciculus for measures of fractional anisotropy.** In each panel, the leftmost graph shows the beta value over the arclength of the fasciculus, with areas of local statistical significance highlighted in grey. Below that is the percent change in the beta value over time for that tract in the specified comparison for the specific diffusivity measure. The middlemost image in each panel shows an overlay of the p-values on the fasciculus, with regions of statistical significance showing a color other than yellow. Finally, the rightmost panel shows the beta values from the specific comparison with the model of the tract.

**
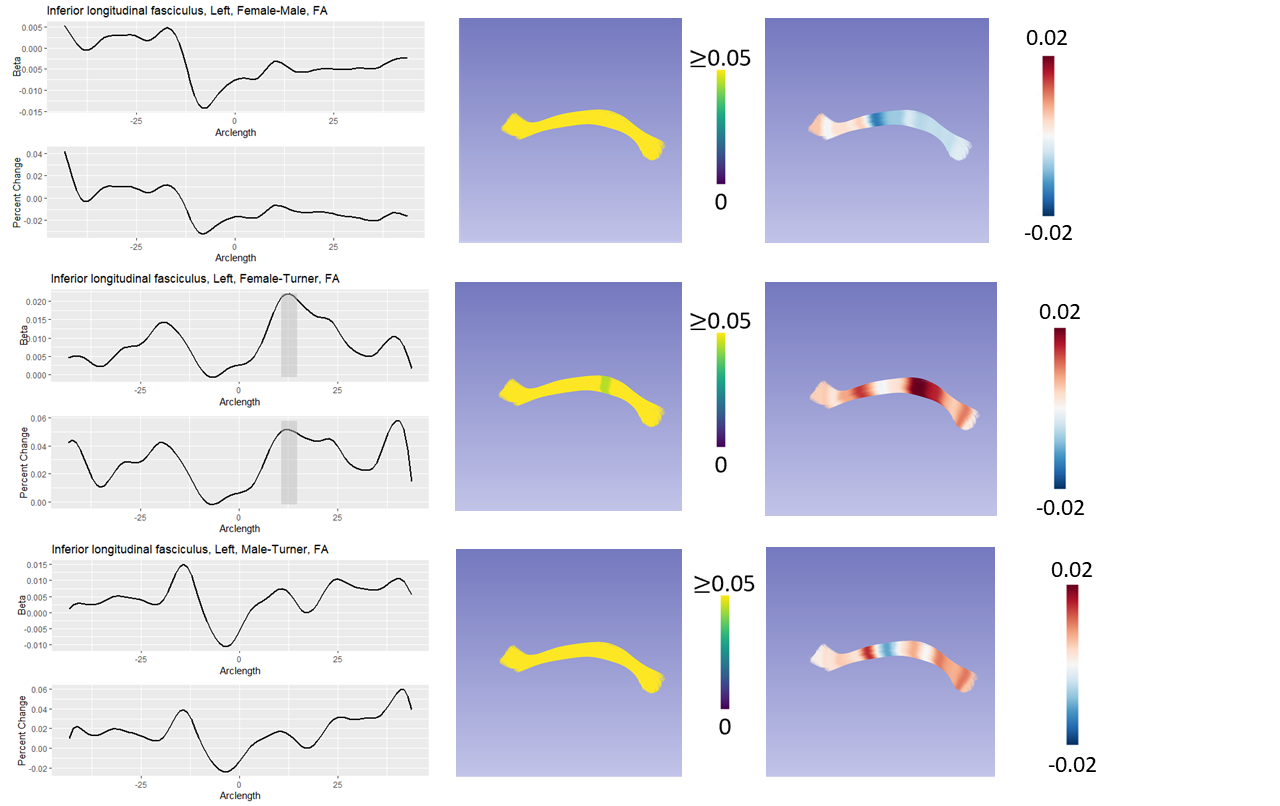
**

**Supplementary Figure 11** **Model of DTI results for the inferior longitudinal fasciculus for measures of fractional anisotropy.** In each panel, the leftmost graph shows the beta value over the arclength of the fasciculus, with areas of local statistical significance highlighted in grey. Below that is the percent change in the beta value over time for that tract in the specified comparison for the specific diffusivity measure. The middlemost image in each panel shows an overlay of the p-values on the fasciculus, with regions of statistical significance showing a color other than yellow. Finally, the rightmost panel shows the beta values from the specific comparison with the model of the tract.

**
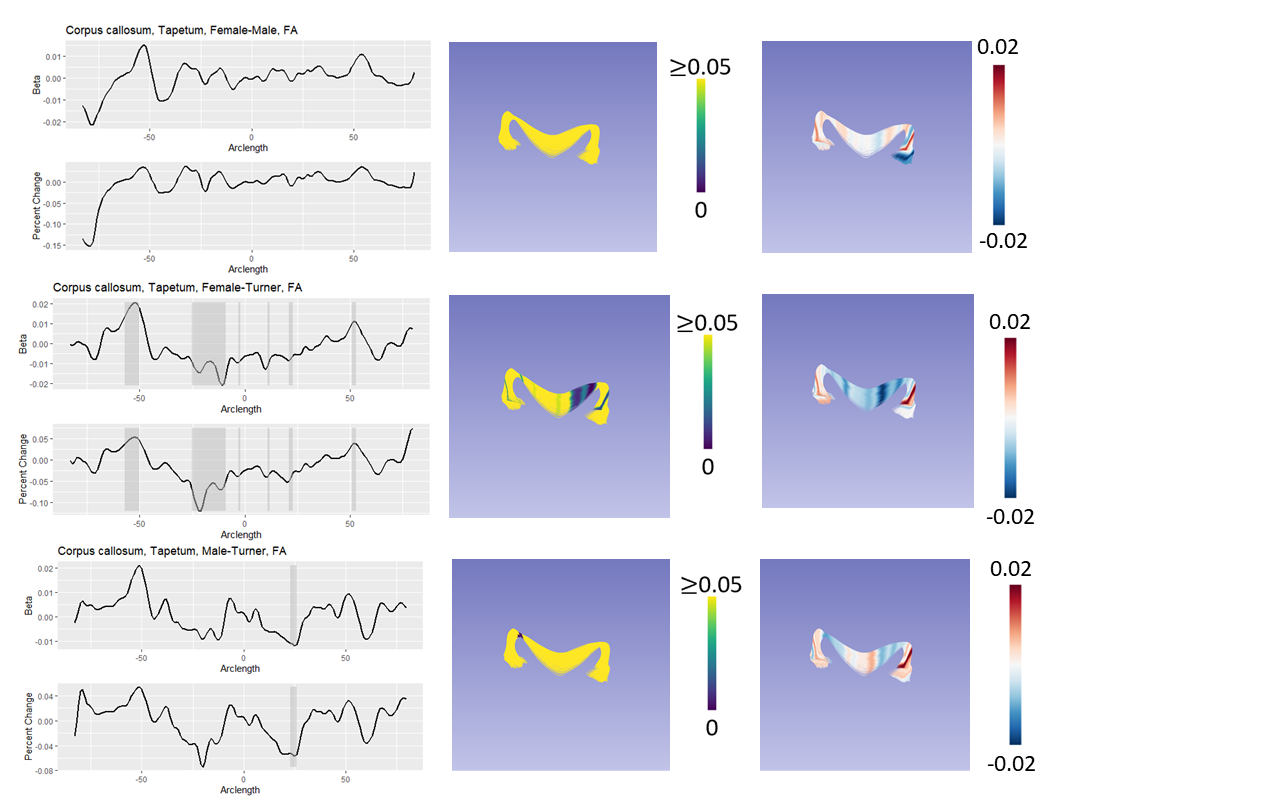
**

**Supplementary Figure 12** **Model of DTI results for the tapetum region of the corpus callosum for measures of fractional anisotropy.** In each panel, the leftmost graph shows the beta value over the arclength of the fasciculus, with areas of local statistical significance highlighted in grey. Below that is the percent change in the beta value over time for that tract in the specified comparison for the specific diffusivity measure. The middlemost image in each panel shows an overlay of the p-values on the fasciculus, with regions of statistical significance showing a color other than yellow. Finally, the rightmost panel shows the beta values from the specific comparison with the model of the tract.
